# Understanding individual neurodegenerative progression in Parkinson’s disease through normative modelling

**DOI:** 10.1038/s41598-025-20484-x

**Published:** 2025-10-21

**Authors:** Charlotte Fraza, Barbora Rehák Bučková, Martin E. Johansson, Rick C. Helmich, Andre F. Marquand, Christian F. Beckmann

**Affiliations:** 1 Donders Institute for Brain, Cognition, and Behavior, Radboud University, Nijmegen, The Netherlands; 2 Department of Medical Neuroscience, Radboud University Medical Center, Nijmegen, The Netherlands; 3 Department of Neurology, Center of Expertise for Parkinson & Movement Disorders, Radboud University Medical Center, Nijmegen, The Netherlands; 4 Department of Neuroimaging, Centre for Neuroimaging Sciences, Institute of Psychiatry, King’s College London, London, UK; 5 Nuffield Department of Clinical Neurosciences, Centre for Functional MRI of the Brain (FMRIB), Wellcome Centre for Integrative Neuroimaging, University of Oxford, Oxford, UK

**Keywords:** Normative modeling, Brain structure, Parkinson’s disease, Disease trajectories, MRI, Neuroscience, Diseases of the nervous system, Parkinson's disease

## Abstract

Parkinson’s disease (PD) is a neurodegenerative disorder with motor symptoms (e.g., bradykinesia, tremors) and non-motor symptoms (e.g., cognitive deficits). Symptom progression varies across individuals, possibly due to differences in the spread of disease pathology. This study investigates individual-level gray matter atrophy in PD patients compared to a reference cohort, modeling neurobiological trajectories to understand symptom progression. Using normative modeling, we mapped individual deviations in gray matter atrophy in PD patients (Personalized Parkinson Project, PPP; N = 408; 42% female) against a reference model (N = 58, 836) of non-diagnosed individuals. Gray matter atrophy was defined as negative deviations from the normative model in cortical thickness and subcortical volume at baseline and two-year follow-up. We correlated the deviations with clinical motor and cognitive symptoms at an individual level and compared changes across PD subtypes (mild-motor predominant, intermediate, and diffuse-malignant). Cross-sectionally, PD patients showed significant gray matter atrophy, which correlated with cognitive impairment. Longitudinally, cortical thinning and subcortical atrophy patterns showed variation amongst subtypes. Specifically, the diffuse-malignant subtype, which is characterized by more diffuse symptoms and faster clinical progression, exhibited pronounced cortical thinning and subcortical atrophy over time. In this paper, we have considered the deviation scores at three levels of granularity: cases vs. control, subtypes, and the individual level. While our findings show subgroup-level patterns of variability, they also provide a method for exploring individual-level metrics of disease progression, acknowledging that individuals may deviate from the predefined categories or groups and can exhibit large variability over time.

**Supplementary Information:**

The online version contains supplementary material available at 10.1038/s41598-025-20484-x.

## Introduction

Parkinson’s Disease (PD) is characterized by considerable heterogeneity in clinical symptoms, neurobiological etiology, and responses to therapy^1–4^. PD is associated with $\alpha$-synuclein accumulation^5^, leading to cell loss in the substantia nigra and (sub-)cortical atrophy^6,7^, underlying the cardinal motor symptoms of bradykinesia, rigidity, and tremors^8^, and cognitive dysfunction^6^. Patients can vary substantially in their severity of motor symptoms^3,9^, cognitive impairment^3^, neuropsychiatric symptoms^8,10,11^, and disease progression^12^, which is hypothesized to be related to different neurobiological etiologies^4^. The marked pathological and clinical heterogeneity of PD highlights the need to consider variations in neurobiology at an individual level, rather than at the group level. While there have been significant advances in our understanding of how PD progresses in the brain for the average or mean patient, understanding the patterns of brain atrophy at an individual level and predicting disease progression across time are key challenges. With this in mind, we aim to map the heterogeneity within PD patients in their brain structural measures both cross-sectionally and longitudinally, creating individualized markers of both current severity and future progression. To achieve this, we employ normative modeling, an increasingly used approach that has been developed in the context of precision computational psychiatry^13^.

Normative modeling is a method that can accurately map the heterogeneity at the population level and use it to make inferences for individuals^13,14^. Through this approach, the mean and centiles of variation for the population can be calculated, enabling the estimation of z-scores or deviation scores for each individual. These models identify specific brain regions in each patient, for instance, where there might be reduced cortical thickness or subcortical volume. Previously, large normative models to understand healthy aging in a reference cohort have been created^15^. These models have shown that within predefined psychiatric and neurologic disorders, large differences in neurobiological deviation patterns could be found between patients with Alzheimer’s disease^16,17^, ADHD^18–20^, autism^21–23^, schizophrenia^24,25^, and participants with genetic mutations or pathogenic copy number variations^26^. For instance, patients with Alzheimer’s showed large variation in cortical thickness outliers, which correlated with worse cognitive function^27^. A study using normative models of brain development examining the long-term effects of adversity revealed widespread adversity-specific morphometric changes related to future anxiety at the individual level^28^.

Various other methods can be used as additional validation and to further analyze the individual deviation scores created with normative modeling, such as clustering or subtyping^18,29,30^. One way to cluster patients and their deviation scores is based on a large set of clinical measurements, including data from motor symptoms, demographic measurements, blood biomarkers, neuropsychological tests, sleep measures, and other non-motor symptoms related to PD^12,31^. This method classifies PD patients into three distinct subtypes: mild-motor predominant (MMP), intermediate (IM), and diffuse-malignant (DM). The clinical characteristics of these subtypes align with different disease propagation models of PD, i.e., brain-first PD versus body-first PD, where the body-first subtype (overlapping with the DM subtype) is associated with an older disease onset, more diffuse and more bilateral symptoms, and ascending alpha synucleinopathy, whereas the brain-first subtype (overlapping with the MMP subtype) is associated with younger disease onset, more focal and unilateral symptoms, and a descending alpha synucleopathy^32^. Furthermore, these subtypes have been previously validated^12,32^, and shown to differ in progression rate and survival rates^32,33^, and motor-related brain activity^34^.

In this study, we first use normative modeling to map deviations from an expected pattern of cortical thickness and subcortical volume (i.e., ‘neurophenotypic diversity’) in individuals with PD. This approach allows moving beyond between-group comparisons, which could be limiting in light of the marked heterogeneity in the patient population. We expect to find consistent negative deviation scores among patients in subcortical areas, such as the bilateral putamen and left amygdala^7,35^, and widespread cortical atrophy patterns^6^. Furthermore, we hypothesize that, aside from the gross group effects, such as for the subtypes, or differences between patients and controls, there will be subtle individual differences between patients in their cortical thickness and subcortical volume deviation scores that map onto the severity scores of behavioral symptoms, such as impaired cognition or general motor symptoms.

We then model individual-level trajectories of PD longitudinally across time and quantify whether certain patients have a more malignant trajectory, using a recent extension of the normative modeling approach that enables us to estimate significant longitudinal changes based on cross-sectional reference models^24^. Currently, for neuroimaging methods, this is the only method developed to adjust cross-sectional models for longitudinal inferences. Although *true* longitudinal models have been devised for height and weight growth charts^36^, these are currently lacking for neuroimaging features. With this method, we can identify brain areas that show large longitudinal negative deviations, indicating significant atrophy over time, thereby helping to understand how the disease unfolds in individual patients. Finally, we evaluate the degree to which the longitudinal deviations score trajectories of patients with PD map onto the three putative subtypes (MMP-IM-DM)^31^, showing that the defined subtypes converge upon different cortical thickness and subcortical volume trajectories, providing a neurobiological basis for the subtypes.

In summary, the objectives of this study are fourfold: (i) First, we aim to cross-sectionally map the heterogeneity in cortical thickness and subcortical volume for patients with PD compared to a reference cohort at two time points (baseline and 2-year follow-up) using normative modeling. (ii) Second, we examine the correlations between the deviation scores and clinical features relevant to PD, such as impaired cognition and tremor severity. (iii) Third, we try to identify which brain regions show significant longitudinal changes in cortical thickness and subcortical volume in patients with PD, and (iv) lastly, we demonstrate whether clinically defined PD subtypes capture longitudinal neurophenotypic differences.

## Materials and methods

**Fig. 1:**
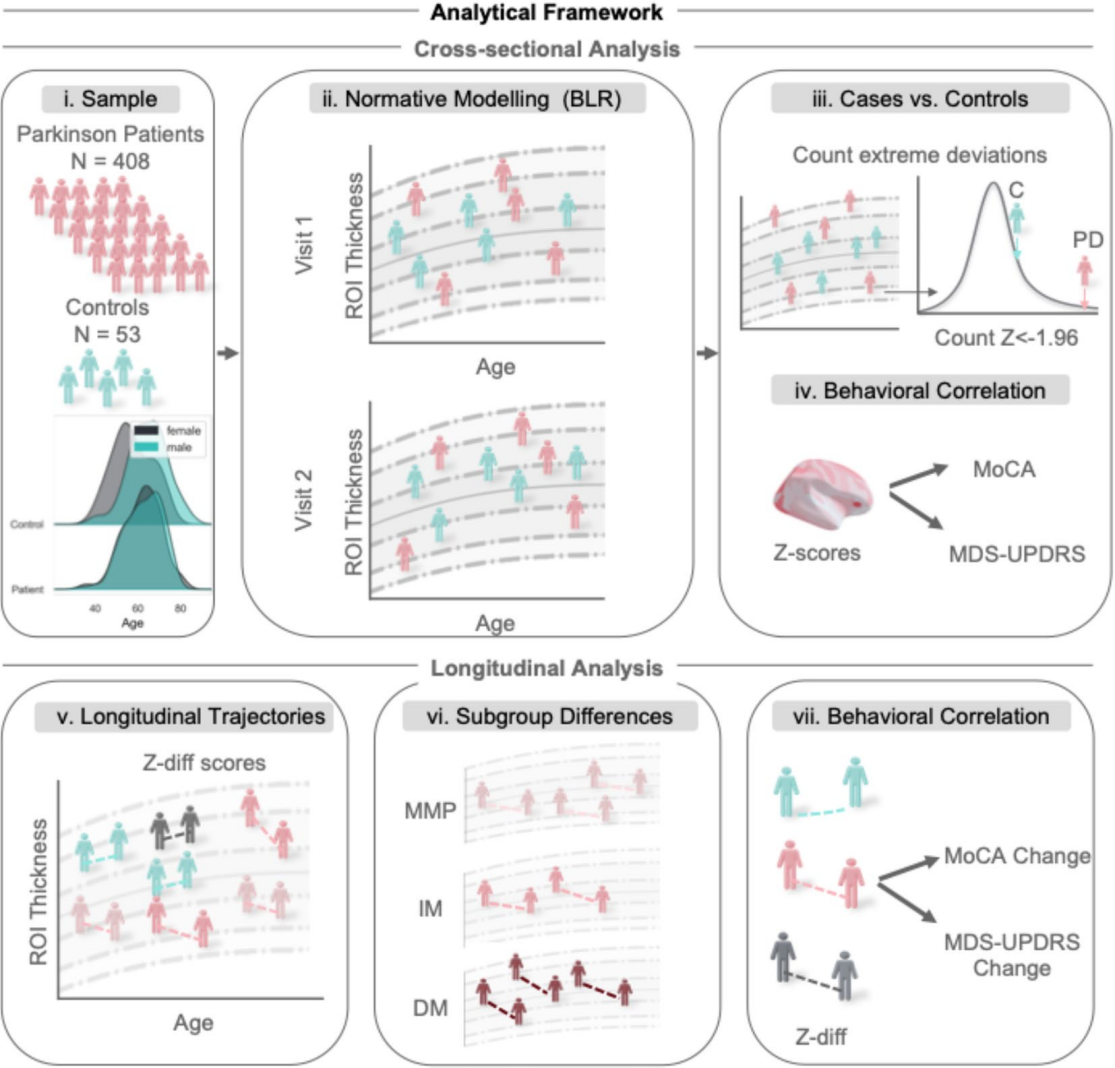
Analytical Framework for Cross-Sectional and Longitudinal Normative Modeling. (**i**) Sample Overview: Overview of the Parkinson’s study sample, showing 408 patients and 53 controls. (**ii**) Cross-Sectional Normative Modeling Framework: Used to estimate population means and centiles of variation on which we estimated the individual deviation or z-scores. (**iii**) Cases vs. Controls Comparison: Based on the total count of extreme negative z-scores (z ≤ -1.96). (**iv**) Correlation with Behavioral Measures: Correlating z-scores and two behavioral measures, for cognition (MoCA) and a total score from the Unified Parkinson’s Disease Rating Scale (UPDRS). (**v**) Different Longitudinal Trajectories: Showing various theoretical longitudinal trajectories. (**vi**) Identification of Subgroup Differences: Showing cortical and subcortical longitudinal differences between the PD subgroups: Mild-Motor Predominant, Intermediate, and Diffuse-Malignant (MMP-IM-DM). (**vii**) Correlation with Changes in Behavioral Scores: Correlating the z-diff scores with the MoCA and UPDRS change scores. Figures created in Python 3.8.2. Icons can be obtained from^48,49^. Brain figure created in Python 3.8.2, using the nilearn package version 0.10.2.

We used two datasets for the normative modeling framework: (1) a quality-controlled clinical sample with controls from the PPP study, a longitudinal PD sample collected at Radboud University Medical Center^37^, and (2) a reference dataset, based on several open-source neuroimaging datasets, established previously, to provide the mean and centiles of variation in cortical thickness and subcortical volume across the lifespan^15^. Figure 1 provides an overview of the PPP sample and the analytical pipeline used in this study.

### PD sample description

The PPP is a representative real-world, deeply phenotyped longitudinal clinical sample of patients with PD (N = 408, after exclusions) and healthy controls (N = 53), which includes, amongst others, various PD-related tests (e.g., measures of PD motor and cognitive dimensions), and a comprehensive set of MRI scans. The patients included in the study were within ≤ 5 years since diagnosis, with an age range of 31–82 at time point 1 (baseline) and 33–84 at time point 2 (two-year follow-up) (see Tables 1 and 2 for an overview of the sample of patients and controls). All the data collection of the PPP sample prior to this study was carried out according to the guidelines as defined in the Declaration of Helsinki, the Dutch Personal Data Protection Act, and the European General Data Protection Regulation (GDPR). The study was approved by the Commissie Mensgebonden Onderzoek Region Arnhem-Nijmegen (reference number 2016–2934; NL59694.091.17). Written consent was obtained for all participants^37^. As part of the larger study, MRI scanning was performed at baseline and at 2-year follow-up; the details of the image acquisition parameters can be found below. More detailed information on the PPP can be found elsewhere^37^.

**Table 1:** Demographics and clinical scores of PD patients at visits 1 and 2.

PD Patients (N = 408)	Visit 1	Visit 2
Age (years)	60.97 (8.72)	62.97 (8.70)
Sex (F/M)	175/233	175/233
Years of education	17.18 (4.07)	17.18 (4.07)
MoCA	27.02 (2.18)	25.99 (2.67)
Cognitive composite	0.08 (0.56)	0.08 (0.56)
Disease Duration (Months)	31.68 (17.64)	59.63 (18.60)
Hoehn and Yahr-stage	41 /321/44/2	3/409/45/7/1
LEDD (mg)	526.90 (295.54)	748.05 (393.02)
MDS-UPDRS III: OFF-state assessment		
Total	31.75 (12.14)	37.76 (13.24)
Bradykinesia	15.92 (7.11)	19.03 (7.30)
Rigidity	6.23 (3.19)	7.92 (3.72)
Resting tremor	2.38 (2.72)	2.96 (3.21)
Action tremor	2.34 (2.08)	2.07 (1.84)
PIGD	1.95 (1.32)	2.04 (1.45)

Values are presented as mean (standard deviation) or counts. MoCA: Montreal Cognitive Assessment; LEDD: Levodopa Equivalent Daily Dose; MDS-UPDRS III: Movement Disorder Society Unified Parkinson’s Disease Rating Scale Part III; PIGD: Postural Instability and Gait Difficulty. Cognitive composite was calculated using the mean across z-scores adjusted for age, sex, and education, from tests in six cognitive domains^34^.

**Table 2:** Demographics controls visits 1 and 2.

Controls (N = 53)	Visit 1	Visit 2
Age	60.36 (9.84)	62.36 (9.84)
Sex (F/M)	25/28	25/28
Years of education	16.13 (3.24)	16.13 (3.24)
MoCA	27.66 (1.74)	27.30 (1.86)

Values are presented as mean (standard deviation) or counts. MoCA: Montreal Cognitive Assessment.

### Image acquisition

All scans were acquired using a Siemens MAGNETOM Prisma 3T equipped with a 32-channel head coil. T1-weighted (T1w) anatomical images were acquired using a magnetization-prepared rapid gradient-echo sequence [repetition time (TR)/echo time (TE)/ inversion time (TI) = 2000/2/880 ms; flip angle = 8°; voxel size = 1.0 × 1.0 × 1.0 mm; slices = 192; field of view (FOV) = 256 mm; scanning time = 5 min).

## Reference sample description

We employed a pre-estimated lifespan model that is accessible on GitHub under the name “lifespan_58K_82_sites” – at https://github.com/predictive-clinical-neuroscience/braincharts/tree/master/models – with detailed information on the used neuroimaging samples provided in^15^. The pre-estimated normative models were derived from 58,836 subjects scanned at 82 sites across the ages 2–100, using cortical thickness measures from the Destrieux atlas (148 regions) and subcortical volume measures (37 regions) from the Freesurfer subcortical parcellation. When we focus specifically on the age range of the PD sample, which peaks around 60–70, see Fig. 1, and consider the coverage of the original sample as curated by Rutherford et al. (2022)^15^, we find a very good representation in the 50–80 age range, which is precisely the range of interest in our study. The models incorporated several covariates, age, sex, and fixed-site effects to account for the variability in these factors. The subjects included were selected to represent a broad population sample. The modeling details used in the study are further explained below, in the ‘Cross-Sectional Normative Models’ subsection.

### Cortical thickness estimation

For all the T1w images of the PPP sample, we estimated the cortical thickness using the Destrieux atlas (148 regions) and the subcortical volume area using FreeSurfer’s (version 7.3.2) subcortical parcellation (37 regions)^38^. The T1w images were preprocessed using FreeSurfer’s recon-all method in the ‘longitudinal’ mode, for better consistency between visits^24^. We started with data from 457 patients and 56 controls. We visually quality-controlled all the T1w images, excluding subjects with large artifacts. Additionally, we calculated the Freesurfer Euler Characteristic (EC), which is a reliable measure of scanner quality based on several topological features^39^, and removed subjects with a site-normalized EC above five. This cut-off was established based on the EC histogram^40^. 18 participants were removed during the QC based on the Euler number. Further, participants were excluded if they did not have *both* time points, removing another 34 participants. In total, 408 patients with data at both time points 1 and 2, along with 53 controls, passed the quality control and had data at both time points.

### PD clinical assessments

We used two clinical scores to examine correlations with deviations from brain structure metrics. Motor symptoms were assessed using the Movement Disorders Society Unified Parkinson Disease Rating Scale part III (MDS-UPDRS III)^41^, assessed in a clinically defined off-medication state following > 12 h of dopaminergic medication withdrawal. For the specific items used to derive each MDS-UPDRS III subscale, we refer readers to the original paper^41^. We used the Montreal Cognitive Assessment (MoCA) to measure overall impairment in cognition^42^. For the MoCA score, we used the education-adjusted total MoCA score, where 1 point is added to the raw total score if the participant has 12 years of education or less, following standard scoring guidelines. This score was used for the correlation analysis. The UPDRS score was calculated as the sum total of all Part III item scores (33 scores, items 1–18), including the measures of bradykinesia (11 scores, items 4–9 and 14), rigidity (5 scores, item 3), resting tremor (6 scores, items 17–18), action tremor (4 scores, items 15–16), and PIGD (5 scores, MDS-UPDRS-III items 10–12 and MDS-UPDRS-II items 12–13), as described in the previous work performed by the team work^32^. For the longitudinal analysis, we used the clinical change score $(timepoint 2 - timepoint 1)$ to characterize symptom progression. We have also considered percentage change score as a subsequent analysis where we have calculated $(timepoint 2 - timepoint 1)/ timepoint 1 X 100$ of which the results can be found in the supplement, see Figure S2. To explore relationships with specific motor symptoms rather than with overall severity, the MDS-UPDRS III score was further divided into sub-scores for bradykinesia, rigidity, resting and action tremor, and postural instability and gait disturbances (PIGD).

### PD clinical subtypes

For the clinical subtype analysis, our study investigated the longitudinal trajectories of changes in brain structure for three distinct subtypes of PD^12^: MMP (N = 191), IM (N = 135), and DM (N = 39), collectively denoted as MMP-IM-DM. Patients with substantial missing data in one or more domains across different subtypes were classified as undefined. It was decided to include the undefined subtype in the analysis as the absence of data could stem from non-random missingness. For instance, patients with a greater amount of missing data might exhibit more severe brain phenotypes and larger brain deviation scores. These subtypes were established at baseline based on symptom severity across four clinical domains: motor symptoms, cognitive impairment, rapid eye movement sleep behavior disorder, and autonomic dysfunction, and adjustments were made to account for the impact of disease duration^12,31^. The full clinical characteristics of these subtypes and how they were generated are described at length in^32,43^.

### Cross-sectional normative models

PCNtoolkit version 0.28 was used for all the modeling steps, and a full guide to the method is available as a tutorial at: https://github.com/predictive-clinical-neuroscience/braincharts. The mathematical aspects of the pre-estimated normative models can be found at^15,44^. Briefly, we will explain the different modeling parameters and considerations employed to create the pre-estimated normative models for the thickness and volume measures. We used Bayesian Linear Regression (BLR) with a likelihood warping to predict the response variable ${y}_{n}$ for each subject:

1 $$y={{\varvec{w}}}^{T}\phi \left({\varvec{x}}\right)+{\epsilon }_{s}$$

With ${\varvec{w}}$ the estimated vector of weights and ${\epsilon }_{s}=\text{N}(0,{\beta }_{s}^{-1})$ a Gaussian noise distribution for site $s$, with mean zero and a noise precision term ${\beta }_{s}$ (i.e., the inverse variance)^15,44^. In our case, the outcome measure per subject $\left({y}_{n}\right)$ is the cortical thickness or subcortical volume per ROI. For the covariates $(x)$, we used age, sex, and site. The likelihood warping $\varphi \left({y}_{n}\right)$ is used to model the non-Gaussianity of the data, by warping the non-Gaussian response variable to a Gaussian latent space, for details see^44,45^. The site effects were modeled using a fixed effects setting, which can reduce the bias introduced by variations between different data collection sites. To capture the nonlinearity of the data, a common cubic B-spline basis expansion $\phi \left({x}_{n}\right)$ was used. The hyperparameters were determined by minimizing the negative log-marginal likelihood using the Powell optimization. The z-scores (or deviation scores) were estimated for each subject and ROI according to:

2 $$z_{nd} = \frac{{y_{nd} - \hat{y}_{nd} }}{{\sqrt {\sigma_{d}^{2} + \left( {\sigma_{*}^{2} } \right)_{d} } }}$$

With $\hat{y}_{nd}$ the predicted mean for each subject ($n$) and ROI (d), ${\sigma }_{d}^{2}$ the estimated noise variance per ROI, reflecting uncertainty in the data, and ${\left({\sigma }_{*}^{2}\right)}_{d}$ the variance related to modeling uncertainty. The means and centiles of variation of the pre-estimated normative models have to be adjusted with an adaptation set. This is done by splitting the control group of the PPP sample with a 50–50 split for adaptation and testing. Based on a previous study, this split is large enough for accurate adaptation^46^. However, if one has a larger control sample, it could be beneficial to keep more controls for testing and use a different data split.

A summary measure of the deviation scores was made by looking at extreme deviations, which we defined as values above the 2.5th centile in the lower tail and the 97.5th centile in the upper tail of a standard normal distribution $\left( {\left| z \right| \ge 1.96} \right)$, which indicate infra- or supra-normal brain thickness or volume. We performed a cases-vs-controls group difference test in the number of extreme deviations based on the extreme count measures, using the Mann–Whitney U test.

To estimate the correlation between the z-scores and the behavioral parameters, we reduced the dimensionality of the z-scores through Principal Component Analysis (PCA) and selected the top principal components (PCs) that account for the highest variance, based on the elbow method (see Supplementary Figure S3). We performed the correlation analysis only for the PD patients, as they had all the behavioral measures present. However, in principle, this analysis could also be conducted spanning both cases and controls. Some ‘healthy’ participants can show significant deviation scores in certain brain areas that link to cognitive impairments despite being below a clinical diagnostic threshold. This suggests that some individuals classified as ‘healthy’ can exhibit symptoms of neurological or psychiatric disorders, which could lead to higher brain deviation scores at an individual level, even in the control population, see ^13,47^. Thus, we calculated the Spearman correlation score between the top principal components and the sum scores of the MoCA, and UPDRS, measured only for PD patients, at both time points, whilst controlling for multiple comparisons using the Benjamini–Hochberg false discovery rate (FDR). Afterwards, we have also added an additional correlation analysis to the number of Education Years as a potential factor that could influence the deviation scores.

### Longitudinal normative estimations

We calculated the longitudinal change scores in brain thickness and volume, using the z-diff scores, a metric made to identify significant alterations in brain thickness or volume over time^24^. The z-diff scores are calculated by taking the difference in brain thickness or volume at two time points for each individual and comparing the difference to the variability observed in the control population. Within the longitudinal normative modeling framework, we assume that healthy subjects tend to maintain their position within the population centile over time for short to moderate time intervals. Any large deviation from this expected trajectory suggests a notable change in cortical thickness or subcortical volume. We identified regions with z-diff scores significantly different from zero using the Wilcoxon test, correcting for multiple comparisons through FDR. We mapped areas of extreme longitudinal change, using the total count of extreme z-diff scores (|z-diff|≥ 1.96) for all patients.

For the subtype analysis, the patients with PD were categorized into predetermined subgroups (MMP-IM-DM) in a previous study^12,32^. We examined variations in the extreme z-diff scores among these groups using the Kruskal–Wallis test, a non-parametric statistical method employed to identify significant differences between groups. We conducted Dunn’s test for pairwise comparisons with Bonferroni correction. To determine which brain ROIs were most affected by PD, we visualized where the extreme z-diff scores were on the Destrieux and subcortical atlas for all three PD subgroups.

For the longitudinal behavioral correlation analysis, we applied PCA to reduce the dimensionality of the longitudinal z-diff scores, selecting the top eight components based on the elbow method (refer to Fig. 7). In general, the longitudinal data show a more gradual decline in the explained variance, as compared to the cross-sectional analysis; thus, we decided to retain the top eight components to capture a broader range of variability. This does increase the number of statistical tests and will affect the number of multiple comparisons corrections needed to be performed. We examined the correlation between these top PCs and the changes observed in various dimensions of UPDRS and the MoCA sum score. We considered each dimension of the UPDRS separately because the overall behavioral change score did not consistently capture changes in specific subdimensions (see Fig. 7 for an overview of the change scores). We used the Benjamini–Hochberg method on the* p*-values to account for multiple comparisons.

## Results

### PD-related structural MRI changes: deviations from the norm (cross-sectional analyses)

**Fig. 2:**
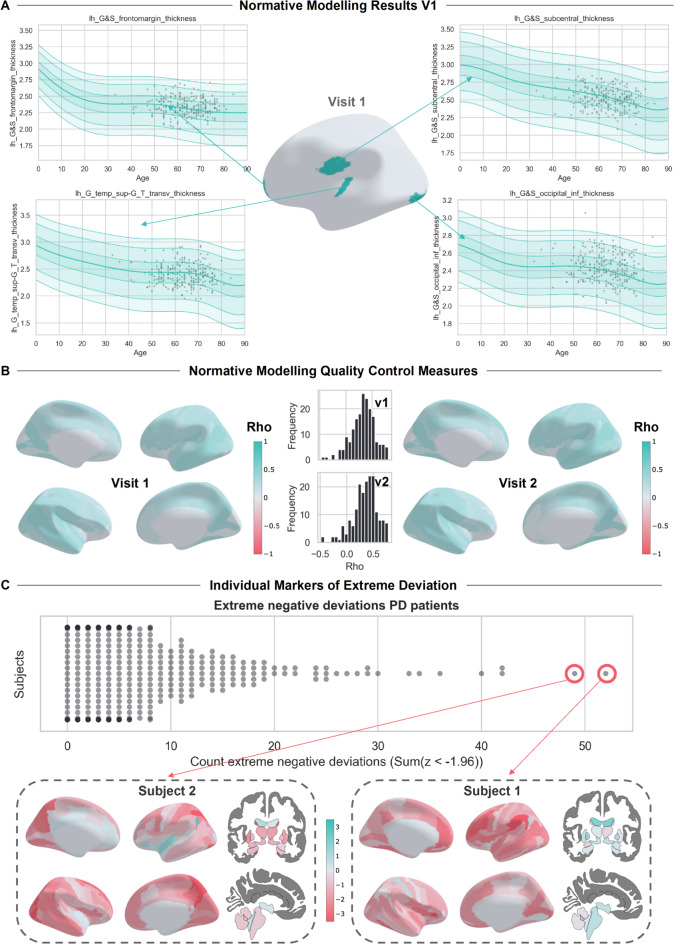
Results of Cross-sectional Normative Modeling for Visits 1 and 2: (**A**) Normative Curves: Estimated using a reference cohort. Overlaid on top is the scatterplot of Parkinson’s patients, highlighted in grey. (**B**) Showing rho (R) for the normative models at visits 1 and 2 in the test dataset for controls. (**C**) Showing individual extreme deviations (z ≤ -1.96) from the normative curve, demonstrating heterogeneity between patients. Figures created in Python 3.8.2, using the nilearn package version 0.10.2.

We show the results of the cross-sectional normative analysis in Fig. 2, displaying the adapted normative models of four ROIs to show the overall fit of the normative models with the new dataset. We observe that the mean and centiles of variation align well with the new dataset (plotted in grey), indicating a successful adaptation of the model. In Fig. 2B, we present plots depicting the correlation between true and predicted thickness or volume scores (Rho/R) for the test set at time points 1 and 2 for the control subjects. The average R at visit 1/visit 2 across all ROIs was 0.33/0.35 (with a maximum value of 0.80/0.78). In Fig. 2C, we show for each individual a measure of atypicality from the norm, as the total count of extreme negative deviations, showing the general heterogeneity between patients. We demonstrate an example for two subjects of how these individualized atypicality scores can be visualized. Subject 1 shows large negative deviations in the cortex, suggesting cortical atrophy (depicted in red), and positive deviations in the brain stem, indicating hypertrophy in the brain stem (depicted in green), as compared to a reference sample. Conversely, subject 2 shows atrophy in the parietal cortex and brainstem, but hypertrophy in the superior temporal gyrus and angular gyrus.

**Fig. 3:**
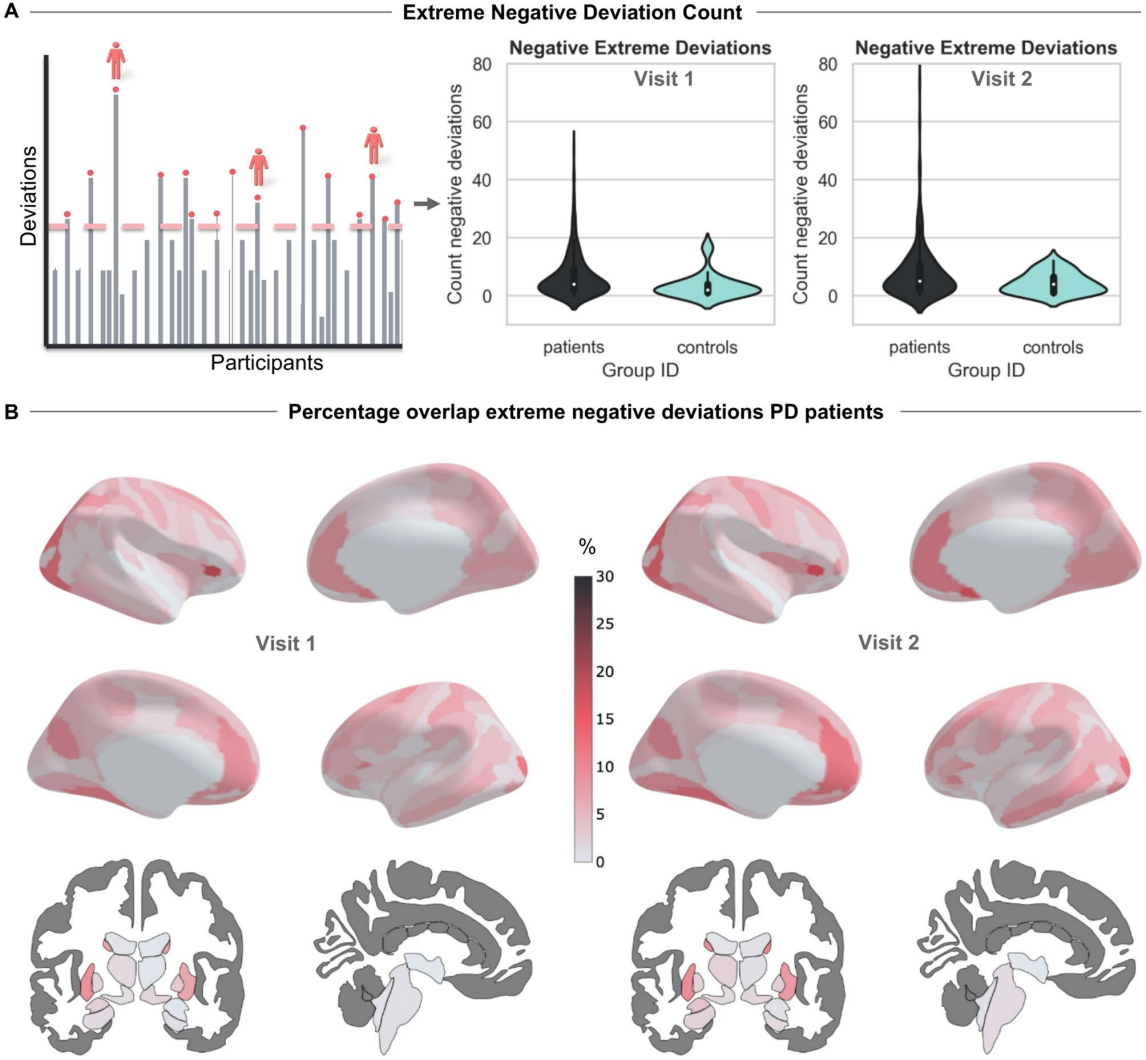
Extreme Negative Deviations from Normative Model in PD Patients at Visits 1 and 2. (**A**) Violin Plots of Negative Deviations: Showing the total count of extreme negative deviations (z ≤ -1.96) between PD patients and controls for visits 1 and 2. (**B**) Overlap Patients Percentage Negative Deviations: Percentage overlap between patients of extreme negative deviations for Parkinson’s patients on the cortical Destrieux and subcortical aseg atlas at visits 1 and 2. Figures created in Python 3.8.2, using the nilearn package version 0.10.2.

We found a significant difference in the total count of extreme negative deviations when comparing patients with PD vs. controls at visit 1 (Mann–Whitney U = 3646.5, *p* = 0.0016, one-tailed) and at visit 2 (Mann–Whitney U = 4313.5, *p* = 0.03, one-tailed), indicating that, patients with PD showed a reduction in cortical thickness and subcortical volume. We found no significant difference in the total count of the extreme positive deviations between patients with PD and controls for visit 1 (Mann–Whitney U = 6605.5, *p* = 0.96, one-tailed) or visit 2 (Mann–Whitney U = 6778.0, *p* = 0.98, one-tailed). The counts of extreme negative deviations are shown in the violin plots presented in Fig. 3A. Figure 3B shows the spatial overlap between patients in their extreme negative deviation scores, as a total percentage of patients deviating in that region divided by the total number of patients, plotted on the ROIs of the Destrieux atlas for both the left and right hemispheres, as well as the subcortical brain regions for visit 1 and visit 2. The highest overlap of extreme negative deviation scores among subcortical ROIs was predominantly observed in the putamen and caudate, whereas the cortical thickness values exhibited pronounced overlap within the occipital and temporal regions. In the supplement, a comparison to the raw score case–control analysis can be found, see Table S1. Furthermore, a detailed list of ROIs with the highest overlap percentages at visits 1 and 2 can be found in the supplement Tables S2 and S3. Finally, to check the potential relationship between disease duration and deviation scores, we performed a correlation analysis between months since diagnosis and the deviation scores. However, this analysis revealed no significant correlations.

Finally, to assess the replicability of our results, we attempted to replicate the full cross-sectional analysis on the Parkinson’s Progression Markers Initiative (PPMI) dataset^50^, using the Baseline and 2-year follow-up data, reflecting the same time period as the PPP dataset. However, this replication analysis was limited by the small number of healthy controls per site in the PPMI dataset, see Table S4 and supplementary Figs. S4, S5 and S6. The controls are needed for effective site adaptation, and most of the sites had fewer than 10 controls per site, meaning less than 8 participants on average for site adaptation, with an 80/20 split for adaptation and testing. This severely limited our possibilities to perform a proper site adaptation of the normative models to the new dataset. The statistical comparisons between patients and controls did not give significant results with the limited site adaptation, see supplementary Figs. S7, S8, and S9. We caution that these findings should be interpreted carefully, as they are most likely influenced by the limited control sample and general site heterogeneity. A more detailed account of the full analysis, preprocessing steps, and results is presented in the supplementary material.

### Multivariate analysis of brain-behavior correlation in PD

**Fig. 4:**
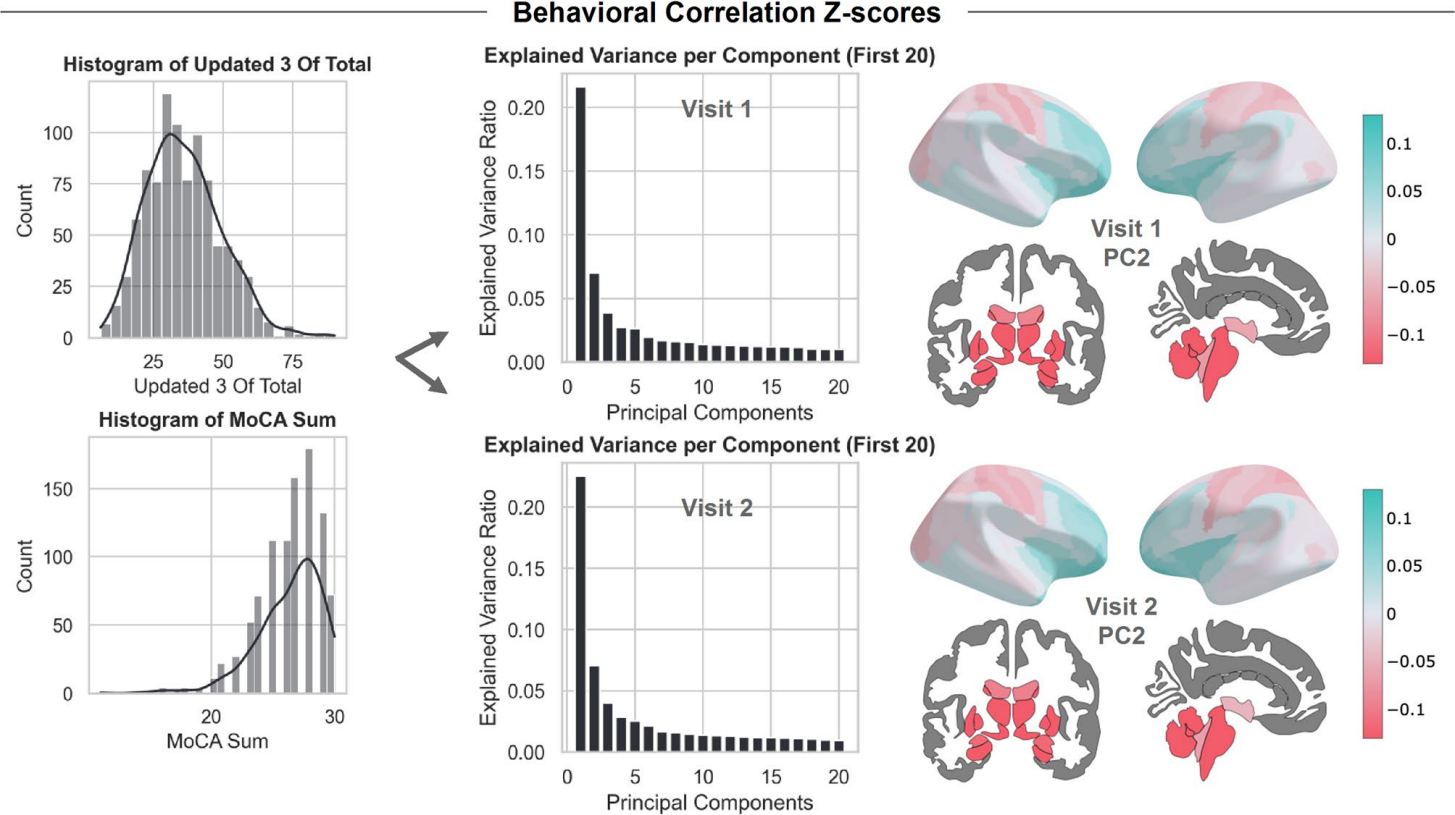
Z-scores and Behavioral Correlation Analysis. We correlated the top three principal components derived from the z-scores with the MDS-UPDRS total score and the MoCA sum score. The explained variance ratio for the first 20 principal components of the z-scores for both visits is plotted. We illustrate the second principal component’s loadings on the Destrieux and subcortical atlas, showing the combined pattern that significantly correlates with the MoCA score. Figures created in Python 3.8.2, using the nilearn package version 0.10.2.

We present an overview of the behavioral correlation analysis using the normative modeling z-scores in Fig. 4. We found a significant (Spearman) correlation between the subject loadings for the second principal component (PC) and the MoCA score: −0.16 (FDR-corrected* p*-value: 0.009) for visit 1 and replicated at visit 2: −0.15 (*p*-value: 0.02). When interpreting the pattern of the second PC, we should consider it as a multivariate signal that combined is correlated with a decrease in the MoCA scores. We can look at the magnitude of the loading of the PC, with a larger loading contributing more to the second PC. To interpret this relationship, we map the loadings of the second PC for visits 1 and 2 in Fig. 3, using the Destrieux atlas for the right and left hemispheres and the Freesurfer subcortical atlas, see supplementary Fig. S1 for the names of the subcortical regions. The second PC shows a multivariate pattern of cortical and subcortical areas with the highest loadings observed in subcortical areas (loading visit 1/loading visit 2): left-putamen (−0.25/−0.26), right-putamen (−0.24/−0.24), left-caudate (−0.21/−0.23), right-pallidum (−0.21/−0.20), right-caudate (−0.20/−0.21), and left-pallidum (−0.20/−0.19). Note that the orientation of the PCs is arbitrary and, as such, cannot be used to directly interpret the relationship between the MoCA score and the z-scores.

We further calculated the correlation with levodopa usage at both visit 1 and visit 2 to account for potential influences of medication on the observed brain deviation scores. No significant correlation was identified in this analysis. Lastly, we correlated Years of Education and our PCs derived from the z-scores for visit 1, which, after multiple comparisons did remain significant with the Spearman correlation between PCA component 2 and Education Years: −0.132 (*p*-value: 0.046).

### Longitudinal brain changes in PD: mapping longitudinal z-diff scores

**Fig. 5:**
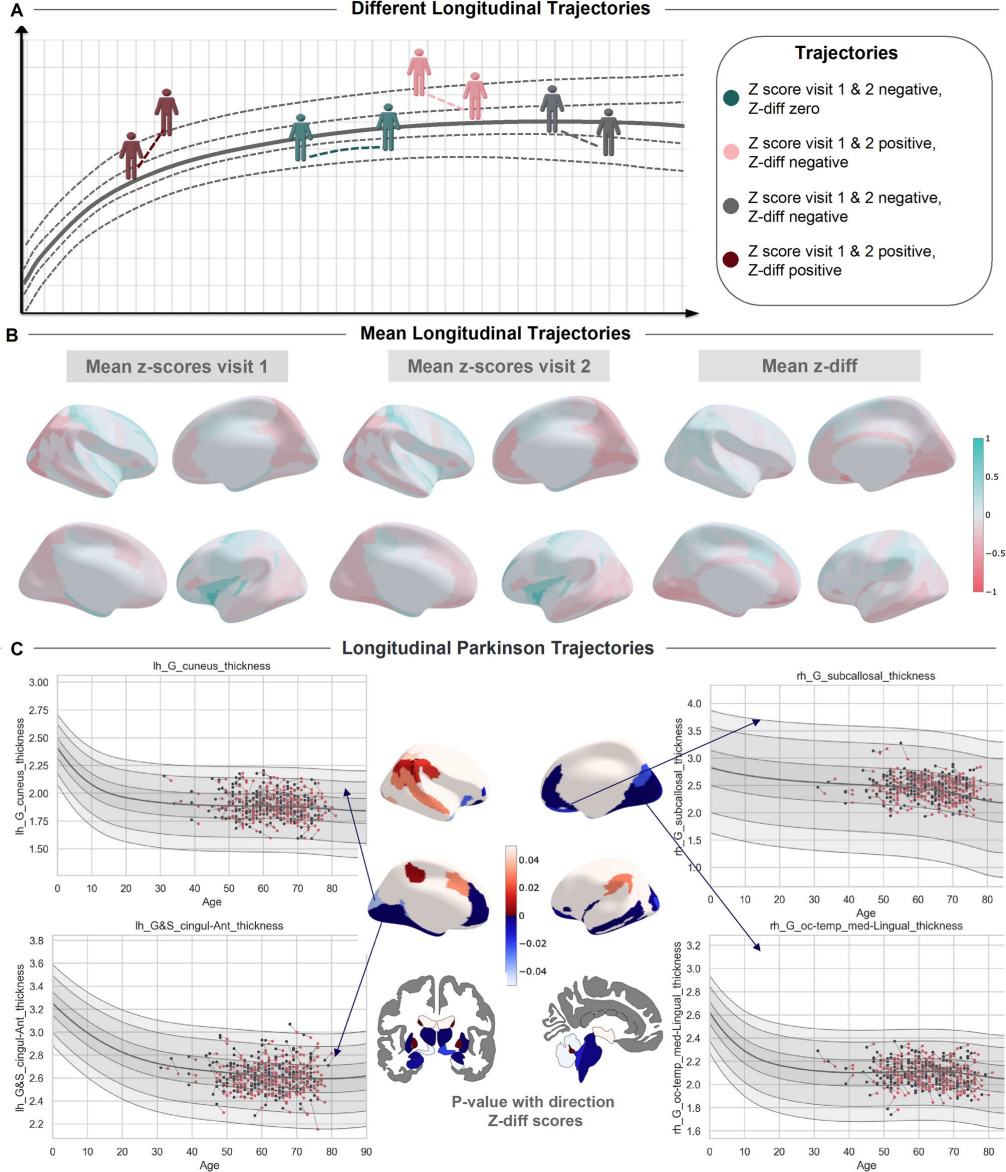
Results Longitudinal Z-diff Scores (**A**) Various longitudinal trajectories are given as an illustration, showing that the interpretation of the z-diff scores changes, depending on the initial z-scores of visits 1 and 2; the colors are for illustration only. The bottom row depicts the mean z-scores for visits 1–2 and the mean z-diff scores. (**B**) The mean z-scores for visits 1 and 2 are plotted, with red and blue indicating positive or negative mean z-scores for the PD group. Along with the mean z-diff scores, with a red color indicating a thickness increase relative to the starting point, and a blue color indicating a thickness decrease relative to the starting point. (**C**) Displaying the signed* p*-values from the Wilcoxon signed-rank test, revealing brain regions where the z-diff scores significantly deviate from zero. The cross-sectional normative models are plotted with a scatterplot overlay of individual patients’ longitudinal trajectories. Brain figures created in Python 3.8.2, using the nilearn package version 0.10.2.

To guide the interpretation of the results, we show the different types of longitudinal trajectories and z-diff scores that are possible during the two visits in Fig. 5. For instance, it is possible to observe negative z-scores for both visits, falling within the same quantile, resulting in a z-diff score close to zero. Conversely, we may encounter two positive z-scores with a downward trend, leading to a negative z-diff score. This would suggest that the patient initially had above-average thickness or volume in that brain region, but exhibited significant atrophy over time compared to the expected growth pattern of remaining within the centile. In Fig. 5A, we plotted the mean z-scores for visit 1 and visit 2, along with the mean z-diff scores, to emphasize the overall longitudinal trend among patients with PD.

For the z-diff scores in Fig. 5B, we illustrated the FDR-corrected* p*-values derived from the Wilcoxon signed-rank tests on the Destrieux and subcortical atlas. The subcortical areas, including the bilateral caudate, bilateral putamen, and bilateral thalamus, exhibited the lowest negative * p*-values, indicating trajectories that diverged from those predicted by the normative model. A full list of significant areas can be found in the supplement. We depicted the two time points as a scatterplot overlaid on the normative models, providing a visual representation of the heterogeneous longitudinal trajectories for patients with PD. These data show that the speed of gray matter atrophy in PD is faster than expected (based on the normative reference models) in certain subcortical areas (e.g. basal ganglia, brainstem, amygdala), as well as cortical areas (e.g. occipito-temporal and anterior cingulate cortex), but slower than expected in posterior parietal cortex, angular gyrus, and posterior cingulate cortex.

### Subtype-specific longitudinal trajectories and behavioral correlates in PD

**Fig. 6:**
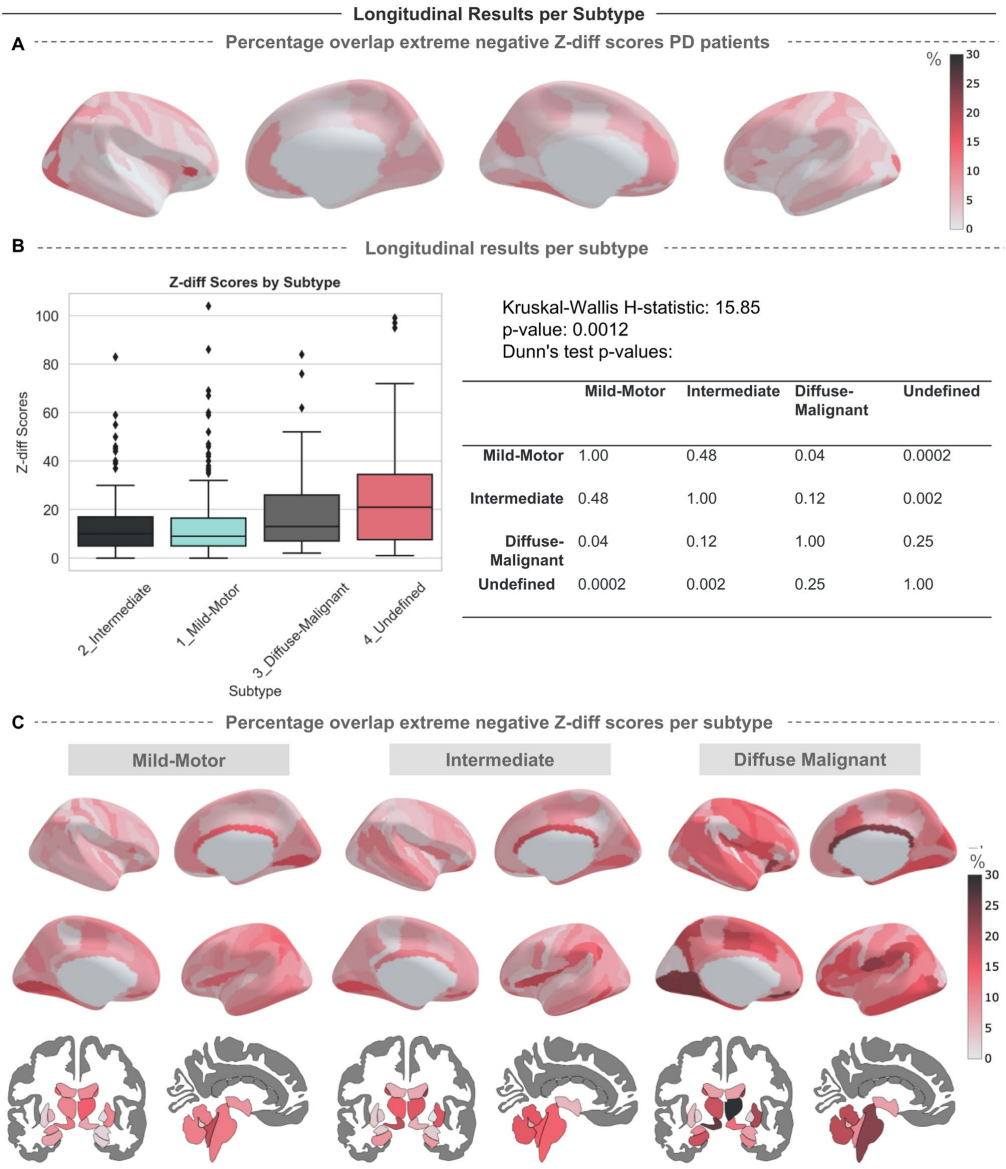
Results Longitudinal Normative Model across Parkinson Subtypes. (**A**) The percentage of overlapping extreme negative z-diff scores among Parkinson’s patients. (**B**) Boxplots depicting the count of extreme negative z-diff scores (z-diff ≤ -−1.96) per subtype, accompanied by the results of the Kruskal–Wallis and Dunn’s tests to assess statistical differences. (**C**) The percentage of overlap in extreme negative z-diff scores per subtype is plotted on the Destrieux and Aseg atlas, showing specific ROIs of the brain where different subtypes exhibit large longitudinal atrophy compared to the healthy population. Brain figures created in Python 3.8.2, using the nilearn package version 0.10.2.

We examined the overlap of extreme values in z-diff scores across all patients with PD to understand how extreme z-diff trajectories are distributed spatially and which brain regions are showing the most longitudinal atrophy, see Fig. 6A. Finally, we evaluate the degree to which the deviations map onto putative subtypes (Fig. 6B). For example, 30% of DM patients showed extreme longitudinal atrophy in the caudate relative to the reference population, compared to around 15% of IM patients and 10% of MM patients. This indicates that the caudate is more frequently affected in the DM patient group. The Kruskal–Wallis test showed a significant difference among the subtypes for the extreme z-diff scores (H = 15.85, *p* = 0.0012). This indicates that there is a difference in atrophy rates between the subtypes. Dunn’s post hoc test showed a significant difference between mild-motor and diffuse-malignant subtypes (Bonferroni-corrected *p* = 0.04). We further observed significant results for the undefined group (the group with missing data), suggesting that the absence of data in the undefined group may not be random and could be driven by clinically relevant markers. For example, the likelihood that individuals with more severe symptoms may have more missing data due to the inability to complete the behavioral tests. Figure 6C shows the extreme z-diff scores for each subtype on the Destrieux and subcortical atlases. The diffuse-malignant subtype exhibits more overlap of extreme negative z-diff scores in the subcortical areas, which aligns with the finding that this subtype has a faster decline in both motor and non-motor symptoms^12,32^.

**Fig. 7:**
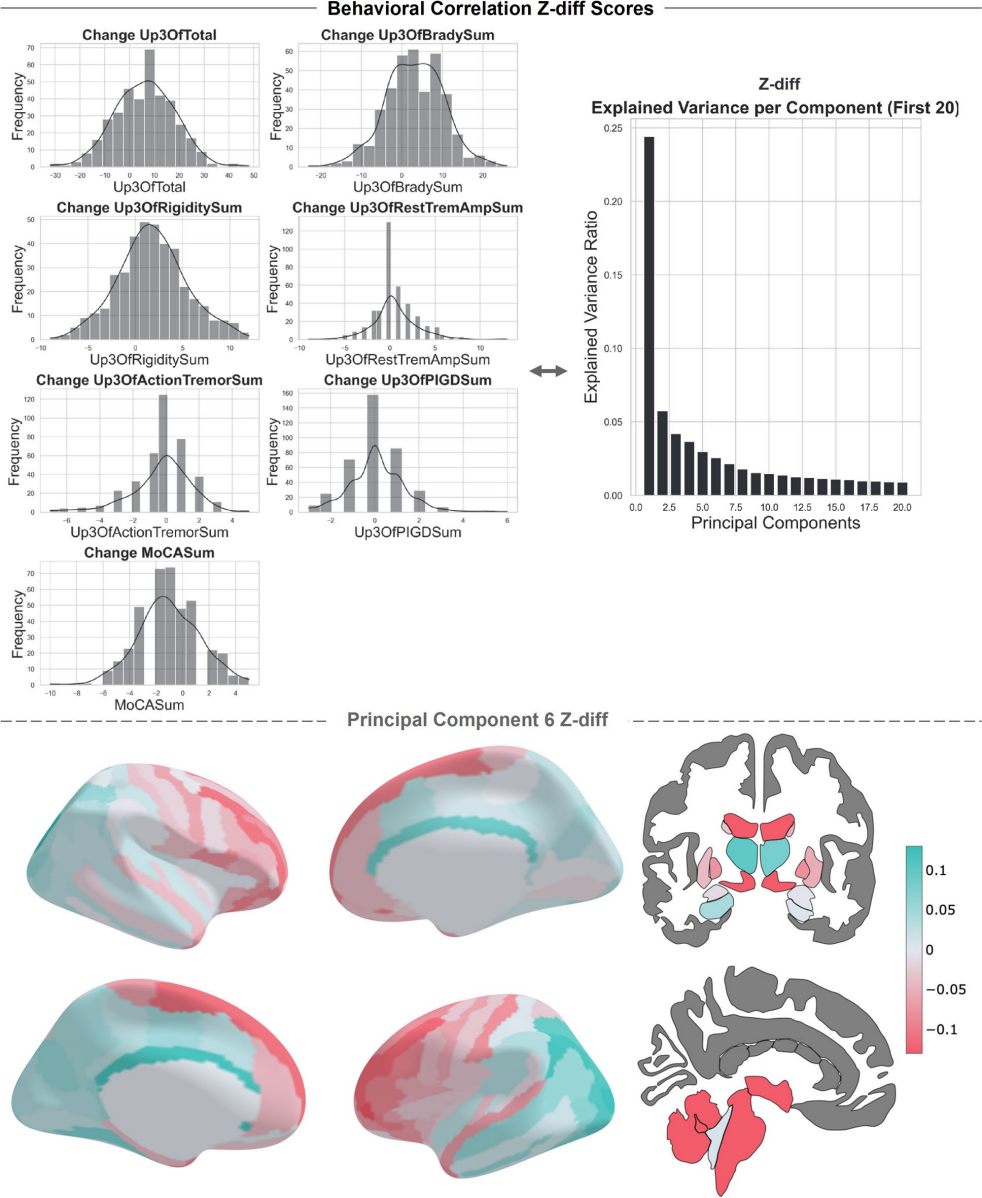
Z-diff and Behavioral Correlation Analysis. Histograms are plotted for all behavioral change measures. The percentage of variance explained by each for the first 20 principal components of the z-diff scores is shown. The loadings of the 6th principal component on the Destrieux and subcortical atlas are plotted. This multivariate pattern has a significant correlation with the change score of the resting tremor severity score. Brain figures created in Python 3.8.2, using the nilearn package version 0.10.2.

We correlated the PCs of the z-diff scores and the changes in the different behavioral subdimensions of the MDS-UPDRS, along with the MoCA sum scores. We identified a significant correlation between PC component 6 and a resting tremor severity score (MDS-UPDRS item 3.17) with a Spearman correlation coefficient of -0.1675 (*p* = 0.048), see Fig. 7. PC6 shows a multivariate combination of areas that together contribute to the significant correlation with the resting tremor severity score. This pattern indicates that larger atrophy patterns in gray matter volume in the multivariate regions are associated with an increase in rest tremor severity.

## Discussion

For this study, we quantified individual trajectories of progressive neurodegeneration in a large sample of PD patients, using a novel approach that compares each single individual with a large reference population. Our findings show: (i) heterogeneous cortical and subcortical deviation patterns in patients with PD, (ii) a correlation between these deviation scores and behavioral features related to PD, (iii) individualized longitudinal atrophy patterns that are related to the changes in clinical features, and (iv) significant longitudinal differences between putative clinically defined PD subtypes. A strength of our approach lies in the ability to analyze brain deviations both at the individual and group levels. For example, Fig. 2 demonstrates individualized cross-sectional normative modeling results, highlighting brain deviation scores for specific participants relative to the population, while Fig. 5 highlights the individualized progression trajectories (z-diff scores) across four brain regions. Using the normative modeling approach, we demonstrated that PD is characterized by heterogeneous reductions in cortical thickness and subcortical volume that, in their combined extreme patterns, are consistent with previous research based on group-level case–control analysis. Moreover, by examining changes in brain structure over two years, we observed PD-related patterns of both atrophy and hypertrophy. Finally, we found that these atrophy patterns differed between putative clinical PD subtypes, for which gray matter atrophy progressed faster for patients within the diffuse-malignant subtype.

A strength of the normative modeling approach is that our results can be interpreted at both the group and the individual level. We can create individualized brain deviation maps, as shown in Fig. 2, alongside the group comparisons through the aggregated deviation scores. This means that we can explore both traditional group-level differences alongside variations in the tails. To illustrate the individualized approach, we highlighted select participants with large deviation scores and show their brain maps in Fig. 2 and in supplementary Figure S8.

Our cross-sectional normative modeling analysis indicated that patients with PD have more widespread reductions in cortical thickness and subcortical volume, whilst also demonstrating large patterns of heterogeneity between patients. When looking at the percentage of overlap of extreme negative z-deviation scores for the subcortical areas, we observed that several brain regions, such as the putamen and the caudate, showed a substantial reduction in volume for patients compared to controls. Previous studies have related the putamen to the motor symptoms present in PD^51^ , and general volume reductions of the putamen and the caudate nucleus have been shown in group-level case vs. control studies^6,52^. For cortical thickness, we observed large overlapping negative deviations in patients with PD in the temporal and occipital lobes. These areas have been correlated with cognitive impairment^53^ with disease duration, and visual deficits in PD^54,55^. However, when looking at individual patients, these exact deviation patterns do not always occur. With the normative modeling approach proposed in this paper, we have the added benefit that we can look at both mean and extreme trends in all deviation scores and individual patterns of deviation, allowing us to look at individual subjects and their deviation patterns in comparison to a reference cohort. This makes the method more flexible to, in addition to individual trends, detect various types of group differences, for example, in the positive or negative tails. This showed that there is large heterogeneity between patients in their atrophy patterns, which remains unaccounted for in typical group-level analyses of atrophy.

With the cross-sectional behavioral correlation analysis, we revealed a significant negative association between the deviation scores’ second principal component and the MoCA sum score. The overall correlation between the individual z-scores and the observed MoCA sum score was small but consistent with our expectations based on previous cross-sectional case–control studies^6^. We can see from the loadings of the second PC on the brain atlases that the subcortical areas and the occipital, temporal, and parietal lobes contribute a large part to this correlation. These areas should be considered as a multivariate pattern that together contribute to the correlation. Cognitive impairment, as measured by the MoCA scores, has been associated previously with changes in cortical thickness and subcortical volume in patients with PD^6,56^. We further highlighted several areas – the putamen, caudate, and pallidum, as exhibiting the highest contributions to the correlation between the z-scores and cognitive impairment, which have been shown in other studies as involved in the cognitive deficits present in PD^57–59^.

In general, for the correlation of the deviation scores to behavior, several factors can be considered, including the magnitude, location, and spatial distribution (sparse vs. clustered) of the deviations. In the current applications of normative modeling, mostly the magnitude of the deviation scores is used for the prediction of clinical severity measures. However, this could be extended to the location and spatial distribution factors as well. For the location, large deviation scores in certain areas of the brain might be more detrimental if these areas or regions are critical for certain processes, and participants with large deviation scores in these regions might have a different clinical profile than those with scattered widespread atrophy patterns. We can consider using a clustering approach as done by ^23^, which, for the case of autism-related deviation scores, clusters into different clinical profiles. In this study, we used the opposite example, where we looked at differences between existing clusters based on clinical scores in their deviation score measures. However, it would be valid to turn this consideration around. Furthermore, we can also consider introducing region-specific weighing if we know that certain regions of the brain have high clinical relevance for the disorder under consideration. For example, weighing the motor regions when considering Parkinson’s disease. However, this would change the currently proposed method, which is more data-driven, to a more theory-driven modeling approach.

Our longitudinal analysis revealed a widespread pattern of cortical thinning in patients with PD and significant negative changes over time in the bilateral caudate, putamen, and thalamus, consistent with findings from cross-sectional results^6^. Furthermore, we observed significant positive z-diff scores (deviation scores over time), particularly in the pallidum. An increase in the relative size of the globus pallidus internus could potentially be linked to heightened inhibitory output to the thalamus, disrupting the balance in the motor circuit, as noted by^60^. Another possibility is that the increase in pallidal volume reflects a compensatory adjustment, mirroring increased dopaminergic uptake in the pallidum to counteract striatal dysfunction^61^. Interestingly, we also identified relative cortical thickening primarily in the parietal cortex. While participants exhibited negative deviation scores in these areas during both visits, the z-diff score was slightly positive. This suggests that despite patients with PD being in the negative centile of deviation at both visits, they converged back to the mean of the distribution. This finding can be understood with emerging evidence indicating a compensatory role of the posterior parietal cortex in PD. For instance, compensatory task-related hyperactivity has been observed in the parieto-premotor cortex of the same PD patients as measured here^43^, in line with meta-analyses on motor-related activity in PD^51,62^. Whether the structural and functional changes in the parietal cortex are linked to a change in lifestyle, or if they reflect implicit network adjustments, remains unclear. For instance, aerobic exercise, which is now regularly advised by clinicians, has been shown to slow down the worsening of motor symptoms in PD^63^, in parallel to attenuation of progressive brain atrophy^64^. Furthermore, other lifestyle factors such as smoking and aspirin intake^65,66^, have also been related to PD symptoms.

Lastly, we examined the extreme z-diff scores based on the subtypes proposed by others^12^, i.e. the MMP-IM-DM criteria. These PD subtypes exhibit distinct longitudinal behavioral trajectories, with the DM subtype showing more severe motor and non-motor symptoms and a faster progression^32^. However, these subtypes have also been shown not to be stable over time with 45% of patients changing subtypes between baseline and follow-up^32^. Our results provide a biological underpinning for these results by showing that the DM and MMP subtypes differ significantly in their cortical and subcortical longitudinal atrophy patterns. The DM subtype displayed more extreme negative deviations, particularly in subcortical brain regions, showing that this subtype has an increased rate of atrophy that maps to the high symptom severity in motor and cognitive domains and faster rates of progression in behavioral domains^12,32^. Overall, the diverse longitudinal trends in neuroimaging phenotypes observed among these subtypes correspond with the distinct behavioral phenotypic trends and provide evidence that these clinically defined subtypes map onto distinct brain atrophy patterns, which supports their biological plausibility. However, when we look at patterns of brain atrophy, even amongst the distinct subtypes, there is prominent individual variation. We have the option to reduce this variability down to subtypes, which could be beneficial when making different treatment strategies, or we have the option to assess an individual’s trajectory independently, tracking intervention effects over time through continuous monitoring.

When applying normative models to a population, several factors have to be taken into account. First, the representativeness of the original normative model in relation to the new sample that is being studied, in this case, the PPP sample with Parkinson’s patients. We want the general normative model to have approximately the same demographics as our sample, as otherwise our normative model could be biased^67^. This can relate both to general aspects such as age and sex, as well as to more specific aspects of the population, such as socioeconomic status and race. Furthermore, one could decide to add additional covariates to the normative model to take into account differences between populations, although a retraining of the reference curves with the additional covariates would be necessary. The selection of covariates in normative modeling depends on the specific research question, i.e., what do we intend to correct our z-scores for. For the pre-estimate models, it is advised to only include essential covariates, as adjusting for too many variables at this stage can remove meaningful variance related to the disorder itself. For example, adjusting for socioeconomic status, which is often associated with psychiatric disorders, can remove meaningful differences in brain development.

One constraint then of using the pre-estimated normative models is that the covariates included in the models are often quite general, e.g., age, sex, and site. It might be the case that researchers want to add more specific covariates, such as total intracranial volume (TIV)^28^ or race^67^, into the normative models. The inclusion or exclusion of certain covariates depends on the scientific question of the study. If the goal is to remove all variance associated with overall head or brain size (or other covariates) from the local parameters, then adjusting for TIV could be useful. However, if the aim is to capture typical variation across age, sex, and site, then TIV itself is part of that variation, see also the discussion at^68^. The key consideration is then not simply what we regress out, but how we interpret the resulting deviation scores. If one wants to detect region-specific deviation scores relative to an individual’s overall head size, TIV should be included as a covariate. Conversely, if one wants to characterize broader patterns of variation, with conditions such as microcephaly or macrocephaly^69^, adjusting for TIV would remove meaningful signal.

Researchers that do wish to control for specific covariates could do this in a two-step approach, by first adjusting the pre-estimated models and then regressing out the specific covariate from the deviation (z-) scores. This would remove the strict statistical interpretation of the z-scores, but it could reduce unwanted covariate effects. In this study, we chose not to regress out the TIV, such that the individual deviation scores reflect all sources of normative variation. A caveat then must be placed on the interpretation of the individual deviation scores, that these are not corrected for head size. Therefore, it is important to relate these scores to behavioral measures, as done in the second part of the study, to see if the deviation scores reflect meaningful clinical measurements. In future work, it will be valuable to have two sets of pre-estimated models and, depending on your question, have TIV included as a covariate, allowing researchers to explicitly choose between models that control for head size and those that capture general variation.

Another point of consideration when applying general normative curves to a new site is the site adaptation. In general, while the site adaptation set in this study is relatively small, the key requirement when adapting the normative reference curve to a smaller dataset is to account for the site-specific effects, such as scanner differences, that might bias the model outputs. The primary goal of site adaptation is to create unbiased z-scores that are centered around zero and with a variance matching the healthy controls^15,24^. To achieve this, we adapt the large-scale reference normative model to a subset of control participants scanned at the same site and with the same scanner as the target clinical group. This adaptation allows us to use the normative model trained on a large, more diverse sample, while still aligning it with the characteristics of the specific site used for the Parkinson’s dataset. In our current implementation using the warped Bayesian Linear Regression model in the PCNtoolkit, this adaptation is done by applying the parameters estimated from the normative reference curve to the new dataset, and adjusting it with a mean offset and variance adjustment at the site level. Another commonly used method for addressing site effects when creating the normative reference curves is ComBat, which can be applied when multiple sites are present in the dataset. This is specifically done during the fit of the reference curves and not during the adaptation to a single new site. However, ComBat does come with some limitations, such as removing meaningful variation relevant to the disease process. One other method for site-adaptation is the Hierarchical Bayesian Regression (HBR) method, which supports federated learning and can account for site effects in a fully Bayesian, multi-level framework^70,71^. One downside of this method is that it is relatively computationally demanding as compared to the wBLR method. For more details and a full rundown of different methods that can be used for the incorporation of site effects in the normative models, readers can refer to^72^.

One constraint of the study is that the study’s longitudinal estimations are limited by the availability of only two neuroimaging time points for the PD patient group. Especially, as the average brain atrophy patterns change over the progressive stages of PD^52^. If we want to make markers of early intervention for certain patients or participants, it would be necessary to also have data points in the prodromal stage of PD. Another consideration is that our sample includes the PD age range (31–82), suggesting the inclusion of genetic PD cases, which may differ from sporadic cases, adding to the heterogeneity of the PD group. Furthermore, when interpreting the exact meaning of the z-diff scores, one has to be cautious, especially when the observed changes are small. In general, it is expected that healthy individuals should approximately stay within their estimated centile over time when using longitudinal tracking over a cross-sectional normative model^24^. A variety of different longitudinal trajectories could be possible for patients. For example, a participant might initially be estimated with a positive z-score at visit 1, indicating a slightly larger brain region compared to the population mean and variance. Subsequently, at visit 2, the same participant could show a negative z-score, signifying a comparatively smaller brain region. Combining the two z-scores would then result in a negative z-diff score. Conversely, another participant could have a negative z-score at time point 1 and a slightly less negative z-score at time point 2, leading to an overall positive z-diff score. When we interpret the z-diff scores, we should also consider the participant’s initial and final positions as compared to the population. In the future, studies could benefit from the inclusion of additional time points, allowing for a more extensive exploration of PD patients’ trajectories over a longer time period.

## Conclusion

We used a representative Parkinson’s disease sample to examine individual deviation patterns from cortical thickness and subcortical volume normative models cross-sectionally and longitudinally, providing us with a quantitative measure of cross-sectional and longitudinal atypicality compared to a large reference population. Several areas, such as the putamen and the caudate nucleus, showed extreme negative deviations for patients with PD, and the degree of deviation correlated with clinical markers of cognitive decline. Subcortical regions showed longitudinally the largest amount of atrophy for the patients with PD, while the posterior parietal cortex showed the opposite pattern and remained relatively intact. Finally, we demonstrate that our examination of longitudinal atrophy patterns was sensitive to clinical subtypes of PD, demonstrating marked differences between diffuse-malignant and mild motor-predominant patients in a widespread subcortical and cortical pattern.

## Supplementary Information

Below is the link to the electronic supplementary material.

Supplementary Material 1

## Data Availability

To maintain the privacy of the patients, the Parkinson’s data is available upon request only. A request can be made for the data to the co-author Rick C. Helmich at rick.helmich@radboudumc.nl. The reference normative models are publicly available for scientific use and can be requested from: https://github.com/predictive-clinical-neuroscience/braincharts. The code for this work is available at: https://github.com/amarquand/PCNtoolkit.
