# Supplementary material for: Understanding individual neurodegenerative progression in Parkinson’s disease through normative modelling
Source: Sci Rep. 2025 Oct 21;15:36659. doi: 10.1038/s41598-025-20484-x (PMC12541093; doi:10.1038/s41598-025-20484-x)
Supplement: Supplementary file 1 — Supplementary Material 1 [file 41598_2025_20484_MOESM1_ESM.pdf]

Supplementary

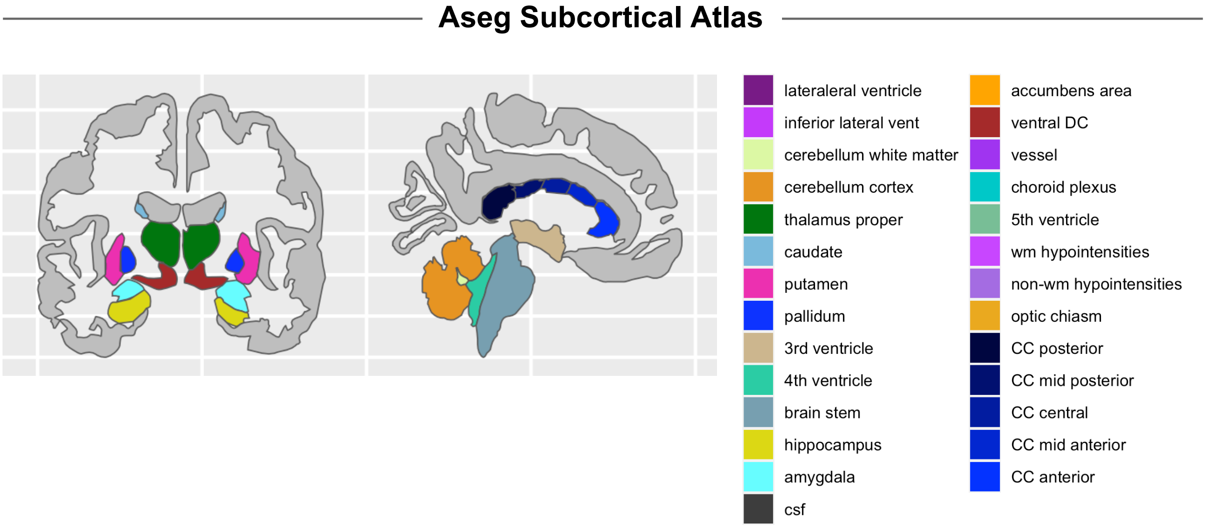

Figure S1 | showing the different subcortical areas of the aseg atlas.

Percentage change scores

We correlated the PCs of the z-diff scores and the percentage change scores in the different behavioral subdimensions of the MDS-UPDRS, along with the MoCA sum scores. We identified no significant correlation between PC components and any of the behavioral percentage change measures. However, we caution readers when interpreting percentage change scores for measures that are not created to be linear and continuous, as is the case primarily for the UPDRS and, to a certain extent for the MoCA scores; these are ordinal scales. This can entail that a one-point change at the lower or higher ends of the scale might correspond to different clinical changes. For example, in the UPDRS, a change from 55 to 56 might reflect a more severe clinical change than a change from 1 to 2. Therefore, in clinical contexts, the raw change score might be more meaningful than a percentage change score.

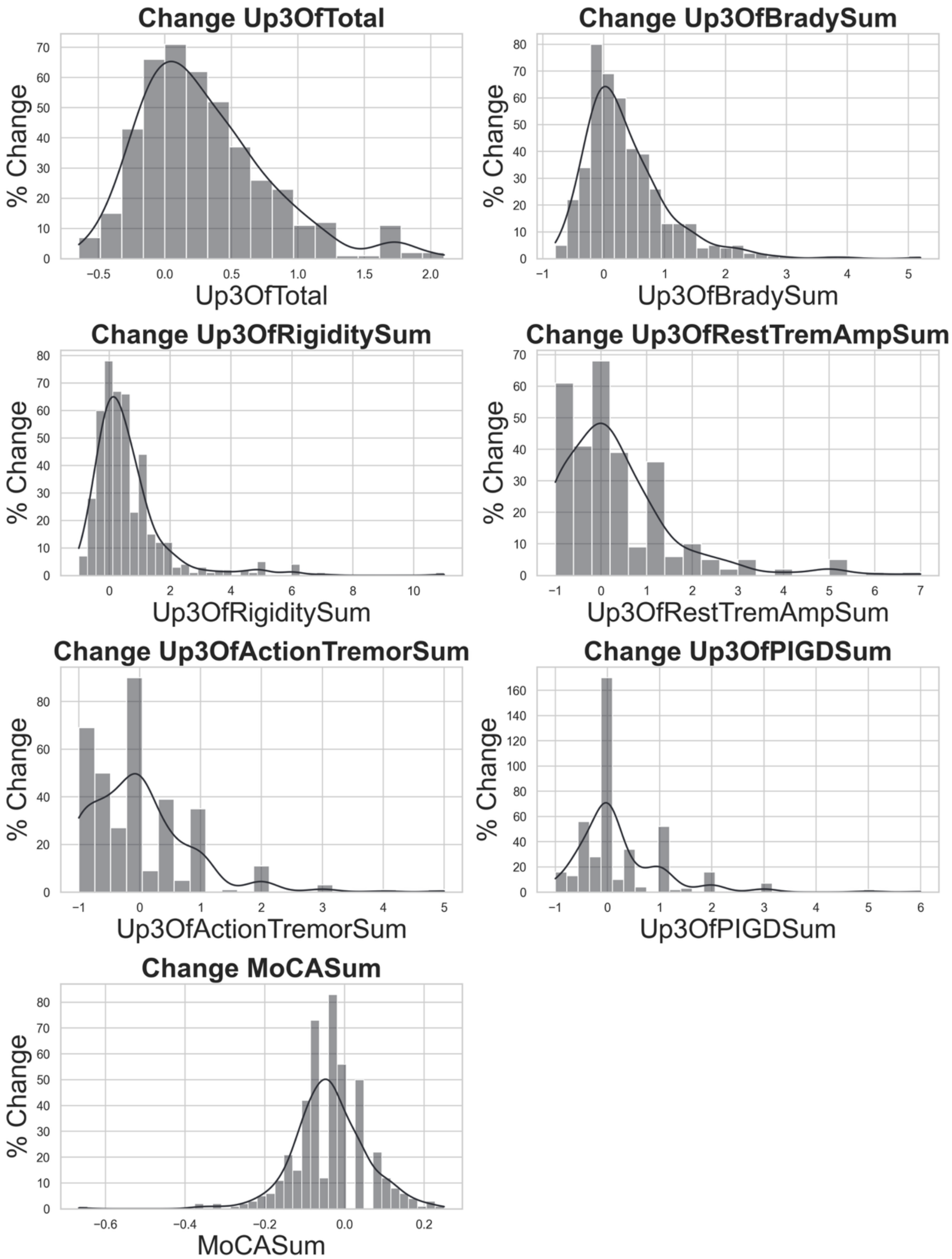

Figure S2 | Behavioral measures percentage change scores. Histograms are plotted for all behavioral change measures in percentage change from time point 1.

Comparison to the raw score case-control analysis

To provide a little better intuition of what normative modeling aims to achieve, we have included a comparison with a basic, yet often flawed, approach using the raw scores and performing a case-control analysis. These raw scores do not take into account covariates such as age, sex, or site. However, if the control sample is carefully matched on these factors, this approach might still be implemented. We compared each IDP between patients and controls at visit 1 using an independent two-sample t-test (assuming

unequal variances), on the raw, uncorrected Freesurfer measures. The resulting p-values were corrected for multiple comparisons using the Benjamini-Hochberg false discovery rate (FDR), and IDPs with an FDR-adjusted p-value < 0.05 were considered statistically significant, see Table S1 below.

A follow-up approach to a simple case-control analysis on the raw scores is to carefully regress out covariates such as age and sex, using, for example, linear regression. Building on this, normative modeling with warped Bayesian Linear Regression allows for the adjustment of age, sex, and site effects in both a linear and nonlinear way, while also accounting for possible non-Gaussian data distributions. Importantly, this method positions individuals in the context of a larger population cohort, allowing us to determine where individuals lie relative to the normative curve. We then obtain covariate-adjusted z-scores. These z-scores enhance interpretability on the individual level as well as reduce the chance of spurious findings, due to the regressing out of specific covariates.

Table S1 | Showing the significant group differences between cases and controls using the raw Freesurfer measures.

IDP	*Name*	*t-stat*	*p-value*	*FDR-adjusted p-value*
156	3rd-Ventricle	5.086616	0.000002	0.000338
166	Right-Lateral-Ventricle	4.011295	0.000125	0.006840
148	Left-Lateral-Ventricle	4.087588	0.000095	0.006840
149	Left-Inf-Lat-Vent	3.965834	0.000148	0.006840
98	rh_G_pariet_inf-Angular_thickness	-3.509579	0.000797	0.029504
92	rh_G_occipital_middle_thickness	-3.287859	0.001561	0.036107
36	lh_G_temporal_inf_thickness	-3.336424	0.001416	0.036107
161	CSF	3.375364	0.001173	0.036107

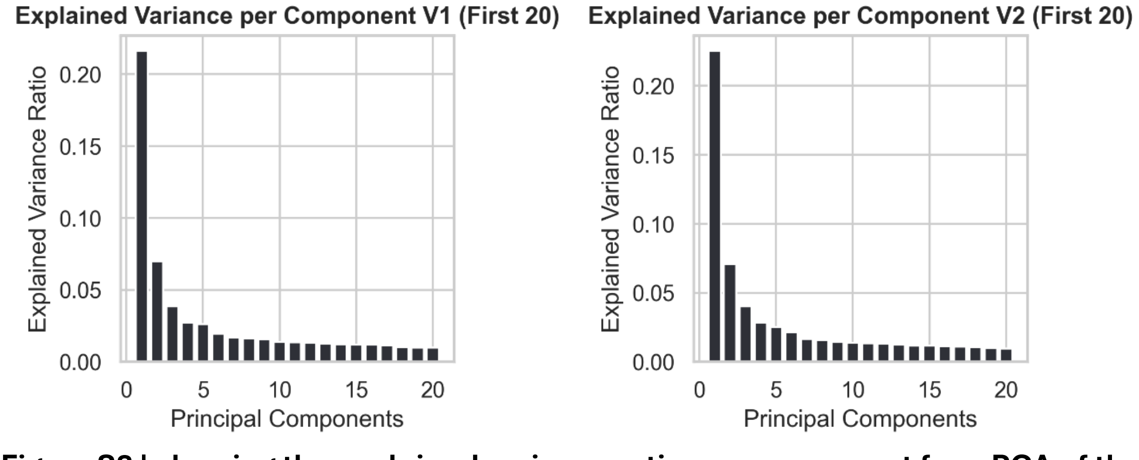

Figure S3 | showing the explained variance ratio per component for a PCA of the z-scores for visits 1 and 2.

Table S2 | Overlap Percentage Negative Deviations Visit 1: Top 10 ROIs with the highest percentage of overlap in extreme negative deviation scores for Parkinson's patients on the cortical Destrieux and subcortical aseg atlas at visits 1.

ROI	*Percentage Overlap*
rh_S_front_sup_thickness	7.35
Left-Caudate	7.60

rh_G&S_cingul-Ant_thickness	7.84
rh_S_intrapariet&P_trans_thickness	8.09
lh_G_subcallosal_thickness	8.09
rh_S_collat_transv_ant_thickness	8.09
lh_S_precentral-sup-part_thickness	8.33
lh_G&S_cingul-Ant_thickness	8.58
lh_Lat_Fis-ant-Vertical_thickness	8.58
lh_S_parieto_occipital_thickness	8.58
Left-Putamen	9.07
rh_G_subcallosal_thickness	9.56
Right-vessel	9.80
lh_S_oc-temp_med&Lingual_thickness	10.29
lh_S_oc_middle&Lunatus_thickness	11.27
rh_G_occipital_middle_thickness	11.52
Left-vessel	11.76
rh_S_oc_sup&transversal_thickness	12.01
rh_G&S_occipital_inf_thickness	12.01
rh_Lat_Fis-ant-Horizont_thickness	16.67

Table S3 | Overlap Percentage Negative Deviations Visit 2: Top 10 ROIs with the highest percentage of overlap in extreme negative deviation scores for Parkinson's patients on the cortical Destrieux and subcortical aseg atlas at visits 2.

ROI	***Percentage Overlap***
lh_G_subcallosal_thickness	8.33
lh_G&S_occipital_inf_thickness	8.33
lh_G_oc-temp_lat-fusifor_thickness	8.33
lh_S_parieto_occipital_thickness	8.58
lh_Lat_Fis-ant-Horizont_thickness	8.82
Right-vessel	8.82
Right-Caudate	9.31
Left-Putamen	9.56
Left-Caudate	9.80
rh_G&S_cingul-Ant_thickness	9.80
rh_S_collat_transv_ant_thickness	10.54
rh_G&S_occipital_inf_thickness	11.03
lh_G&S_cingul-Ant_thickness	11.27
lh_S_oc-temp_med&Lingual_thickness	11.52
lh_S_oc_middle&Lunatus_thickness	11.76
rh_G_occipital_middle_thickness	13.00
Left-vessel	13.24
rh_S_oc_sup&transversal_thickness	13.48
rh_G_subcallosal_thickness	14.46
rh_Lat_Fis-ant-Horizont_thickness	15.20

ROIs with significant negative p-values for the longitudinal analysis:
Left Hemisphere (lh):
[lh_G_cuneus_thickness, lh_S_oc_middle&Lunatus_thickness, lh_G_cingul-Post-ventral_thickness, lh_S_oc-temp_lat_thickness, lh_G_occipital_middle_thickness, lh_S_collat_transv_post_thickness, lh_G_orbital_thickness, lh_G_Ins_lg&S_cent_ins_thickness, lh_Pole_temporal_thickness, lh_S_orbital-H_Shaped_thickness, lh_S_oc-temp_med&Lingual_thickness, lh_Lat_Fis-ant-Horizont_thickness, lh_G_temporal_middle_thickness, lh_G&S_cingul-Ant_thickness, lh_Pole_occipital_thickness, lh_S_orbital_med-olfact_thickness, lh_S_calcarine_thickness, lh_G_oc-temp_med-Lingual_thickness, lh_S_collat_transv_ant_thickness, lh_S_oc-temp_med&Lingual_thickness, lh_Pole_temporal_thickness, lh_S_orbital-H_Shaped_thickness, lh_S_oc-temp_med&Lingual_thickness, lh_G_temporal_middle_thickness, lh_G&S_cingul-Ant_thickness, lh_S_collat_transv_post_thickness, lh_Pole_occipital_thickness, lh_S_orbital_med-olfact_thickness, lh_S_calcarine_thickness]

Right Hemisphere (rh):
[rh_Pole_temporal_thickness, rh_S_circular_insula_ant_thickness, rh_S_parieto_occipital_thickness, rh_G_rectus_thickness, rh_S_orbital_lateral_thickness, rh_S_collat_transv_ant_thickness, rh_Pole_occipital_thickness, rh_S_collat_transv_post_thickness, rh_S_oc-temp_med&Lingual_thickness, rh_G_cuneus_thickness, rh_S_collat_transv_post_thickness, rh_S_orbital_med-olfact_thickness, rh_G_oc-temp_med-Lingual_thickness, rh_S_orbital-H_Shaped_thickness, rh_S_calcarine_thickness, rh_S_orbital_med-olfact_thickness, rh_G_oc-temp_med-Lingual_thickness, rh_S_suborbital_thickness, rh_S_orbital-H_Shaped_thickness, rh_S_calcarine_thickness, rh_S_orbital-H_Shaped_thickness, rh_G_oc-temp_med-Lingual_thickness]

Subcortical Regions:
[Left-Hippocampus, Left-Amygdala, Right-Amygdala, Brain-Stem, Left-Cerebellum-Cortex, SupraTentorialVolNotVent, Right-vessel, Left-Accumbens-area, Right-Thalamus-Proper, SubCortGrayVol, Left-Caudate, Right-Caudate, Right-VentralDC, Right-Inf-Lat-Vent,]

ROIs with significant positive p-values for the longitudinal analysis:
Left Hemisphere (lh):
[lh_S_cingul-Marginalis_thickness, lh_G_pariet_inf-Supramar_thickness, lh_G&S_cingul-Mid-Ant_thickness]

Right Hemisphere (rh):
[rh_S_interm_prim-Jensen_thickness, rh_G_pariet_inf-Angular_thickness, rh_G_pariet_inf-Supramar_thickness, rh_S_temporal_sup_thickness, rh_G&S_occipital_inf_thickness, rh_S_intrapariet&P_trans_thickness]

Subcortical Regions:

[Right-Pallidum, Left-Pallidum, Left-Cerebellum-White-Matter, Right-Cerebellum-White-Matter]

PPMI results

In this study, we tried to replicate the results of the PPP dataset using the Parkinson's Progression Markers Initiative (PPMI) dataset. Below, we give a quick overview of the preprocessing steps and the results obtained using this dataset.

For consistency with the analysis performed on the PPP dataset, we only included subjects with imaging data available at Baseline (V1) and at the two-year follow-up (V2). For the PPMI, more timepoints are available, see https://www.ppmi-info.org/access-data-specimens/data, which could potentially be used for future analysis in the longitudinal progression of Parkinson's. However, here we decided to match the time progression with the PPP dataset (2 years). In cases where multiple scans were available at a given time point, the scan with the highest estimated total intracranial volume (eTIV) was selected for further analysis.

The preprocessing of the T1-weighted structural MRI scans followed the same protocol as applied to the PPP dataset. Specifically, we used the FreeSurfer version 7.3.2 with the longitudinal pipeline, which uses data from both timepoints to reduce within-subject variability. For quality control, we used the site-normalized Euler number and excluded participants with values above the threshold of 5. In total, this removed 5 participants, see Figure S4.

PPMI Normative Modeling

For the normative modeling, we included the covariates age, sex, and site. To ensure that the site-specific adaptation could be done, we only included data from sites with at least 10 or more healthy controls. In general, this threshold is quite low and not recommended. However, as the PPMI dataset did not include a higher number of controls per site, this was the only feasible approach. Ideally, we would recommend including at least 20-50 controls per site for site adaptation, to avoid the risk of individual low-quality or deviating controls disproportionately affecting the normative model. For this analysis, sites that did not meet the 10 controls were excluded from further analysis.

Afterwards, the control cohort was split into an adaptation set and a test set (80/20 split), using stratified sampling by site to ensure enough subjects came from each site for adequate site adaptation. The final dataset included both patients and controls in the test set and only controls in the adaptation set. For visit 1, the dataset included 443 patients and 143 controls. However, again, when we look at the controls per site in the adaptation set, the number of controls remains very limited:

Table S4 | Number of controls in the adaptation set per site for the PPMI dataset.

Site ID	Number of Controls
1013.0	10
1024.0	13
1026.0	16
1028.0	10
1029.0	9
1031.0	12

1033.0	9
1035.0	15
1062.0	10
1063.0	10

These small sample sizes reduce our confidence in the effectiveness of the site adaptation. For visit 2, the dataset included 261 patients and 17 controls, which was insufficient for reliable site adaptation. As a result, we decided not to include the results of visit 2.

The results of visit 1 are presented below in Figures S7 and S8. To show the deviations from the normative model, we defined the extreme deviations as z-scores exceeding the threshold of ($|Z|>1.96$). For each subject, the number of positive and negative extreme deviations across all regions was calculated. These count scores were then used to investigate differences between patients and controls. To statistically assess the differences between cases and controls, we used the Mann-Whitney U test one sided, to test if patients had more extreme positive or negative deviations. These tests did not give a significant result. However, we again urge caution in interpreting these findings, as the limited number of controls per site for site adaptation and testing could potentially impact the generalizability and reliability of the normative modeling outcomes.

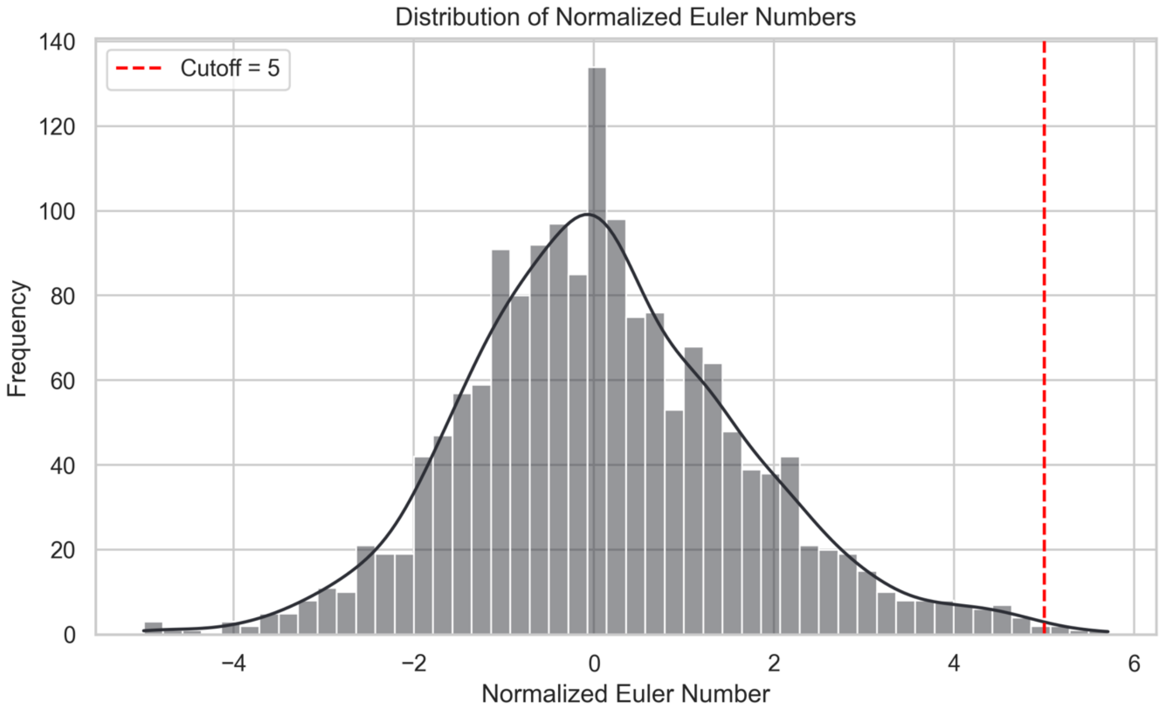

Figure S4 | Distribution of site-normalized Euler numbers across all subjects and all sites. Subjects with a normalized Euler number above the threshold of 5 (marked with a red dashed line) were excluded from further analysis, as these numbers indicate a poor scan quality. Site-specific normalization was done as there can be inter-site differences.

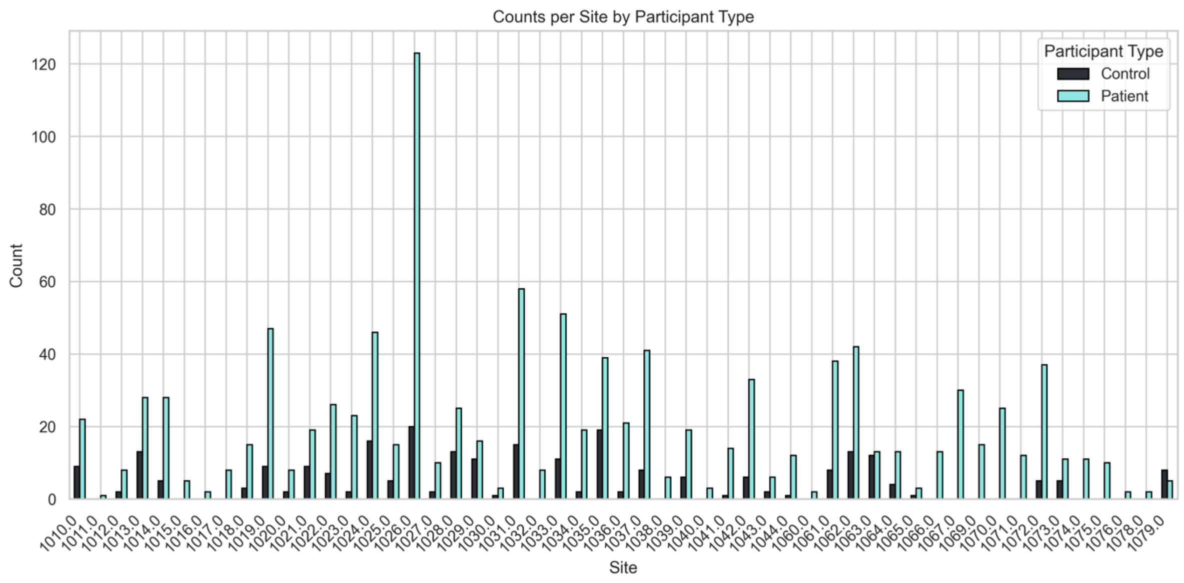

Figure S5 | Sample size per site, separated by participant type (patients and controls in the PPMI dataset). The bars show the number of subjects per site, highlighting the imbalance in participant types across sites.

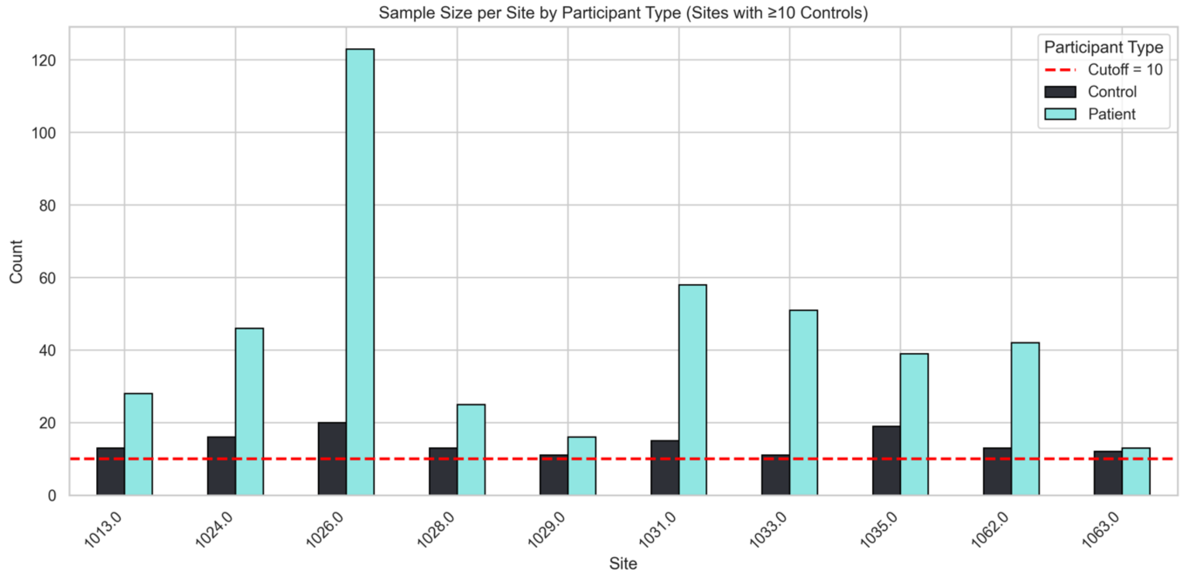

Figure S6 | Sample size per site by participant type (patients and controls in the PPMI dataset) after filtering for sites with at least 10 control subjects for site adaptation.

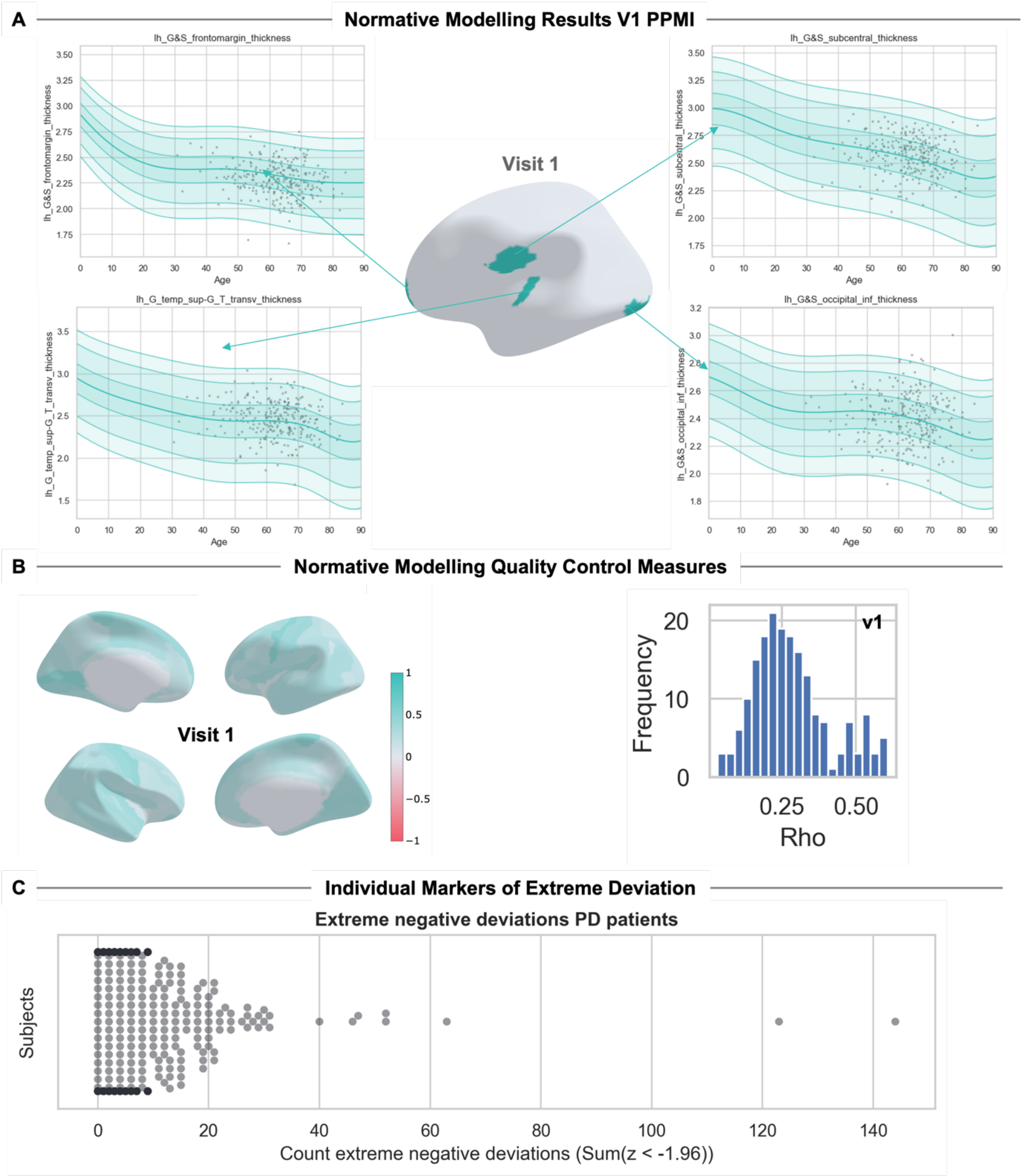

Figure S7 | Results of Cross-sectional Normative Modeling for Visit 1 of the PPMI dataset: A. Normative Curves: Estimated using a reference cohort. Overlaid on top is the scatterplot of Parkinson's patients, highlighted in grey. **B.** Showing rho (R) for the normative models at visit 1. **C.** Showing individual extreme deviations (z ≤ -1.96) from the normative curve, demonstrating heterogeneity between patients.

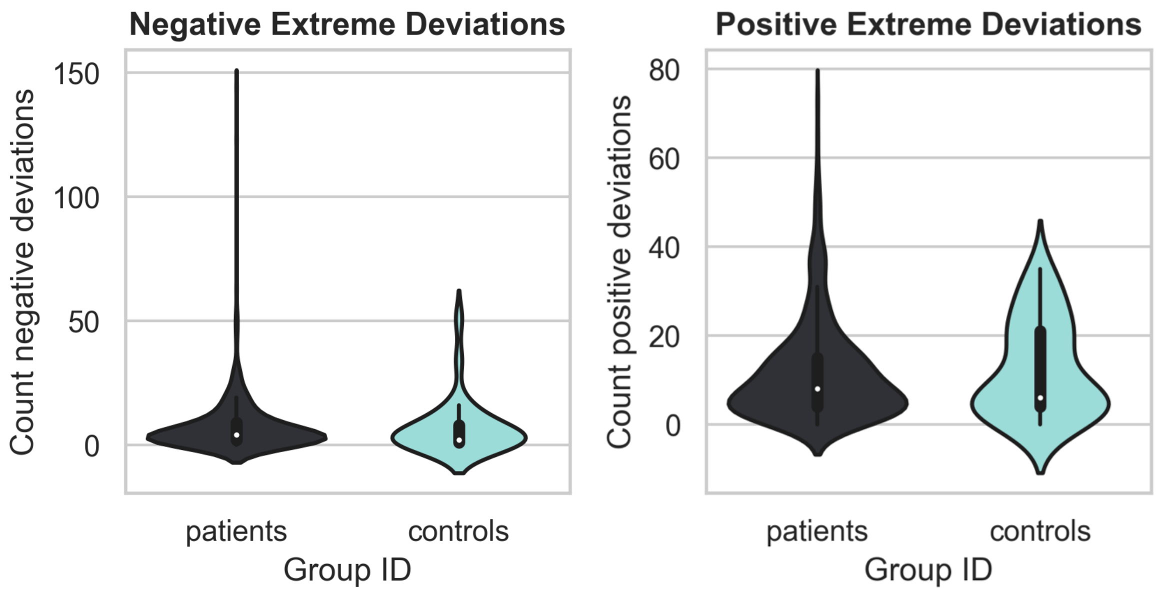

Figure S8 | Extreme Negative Deviations from the Normative Model of the PPMI dataset in PD Patients at Visits 1. (Left to right) Violin Plots of Negative and Positive Deviations, respectively: Showing the total count of extreme deviations ($z \leq -1.96$) between PD patients and controls for visits 1. No significant difference was found between cases and controls, potentially due to poor site adaptation.

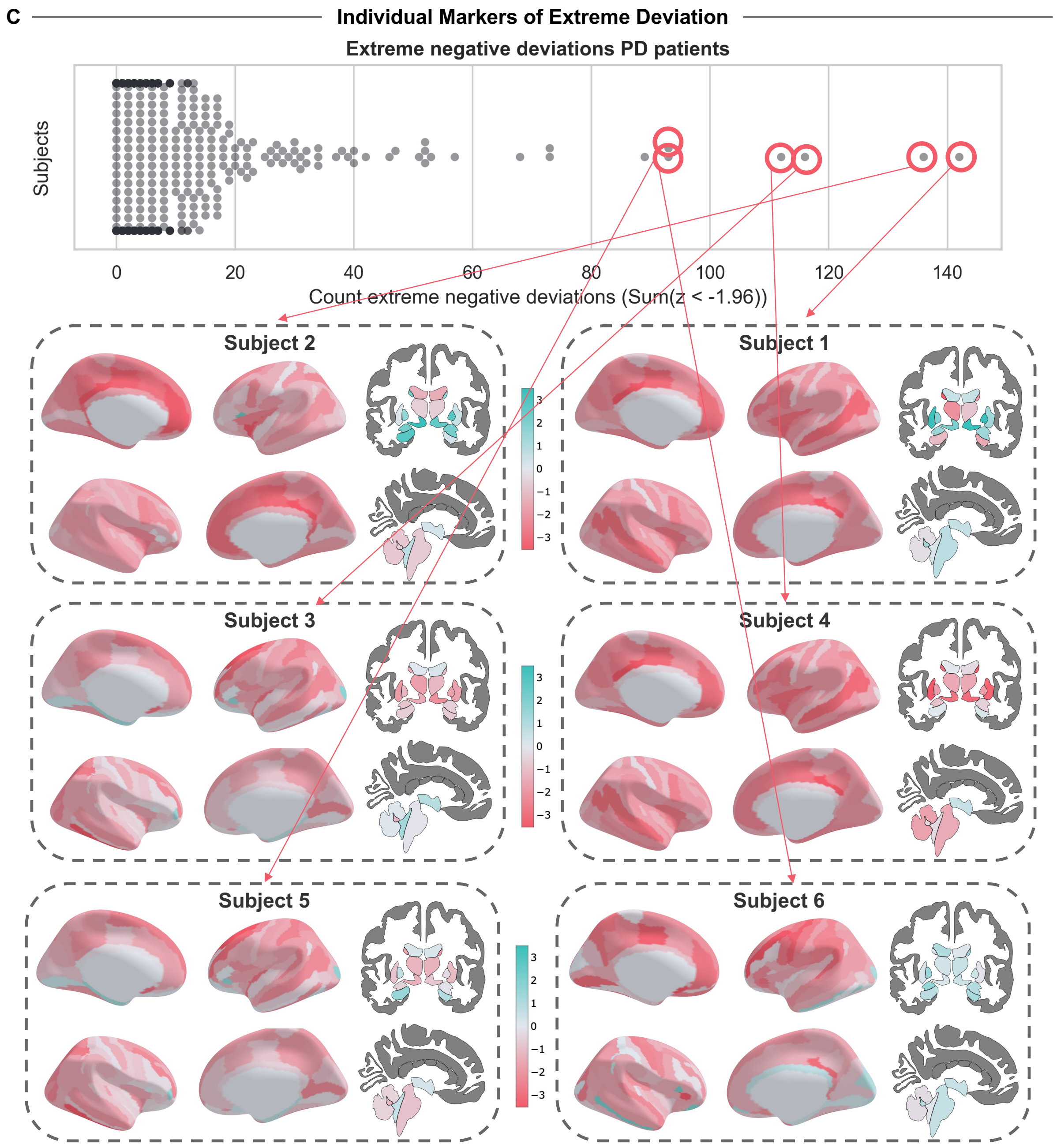

Figure S9 | Results of Cross-sectional Normative Modeling for Visit 1: A. Normative Curves. Showing individual extreme deviations ($z \leq -1.96$) from the normative curve, demonstrating heterogeneity between patients.
